# Introducing standard patient-reported measures (PRMs) into routine maternity care: A pre-implementation qualitative study on women’s perspectives in Finland

**DOI:** 10.1186/s12913-023-09818-5

**Published:** 2023-08-10

**Authors:** Kirsi Väyrynen, An Chen, Riikka-Leena Leskelä, Eija Raussi-Lehto, Reija Klemetti, Seppo Heinonen, Paulus Torkki, Aydin Tekay

**Affiliations:** 1 Department of Public Health, Faculty of Medicine, University of Helsinki, Biomedicum 1, Helsinki, 00290 Finland; 2 Department of Obstetrics and Gynaecology, Central Finland Central Hospital, Hoitajantie 3, Jyväskylä, 40620 Finland; 3 Department of Obstetrics and Gynaecology, Helsinki University Hospital and University of Helsinki, Haartmaninkatu 2, Helsinki, 00290 Finland; 4 Institute of Healthcare Engineering, Management and Architecture (HEMA), Department of Industrial Engineering and Management, Aalto University, Maarintie 8, P.O. Box 15500, Aalto, FI-00076 Finland; 5 Nordic Healthcare Group Oy, Vattuniemenranta 2, Helsinki, 00210 Finland; 6 School of Public Health, Zhejiang Chinese Medical University, No. 548 Binwen Road, Binjiang District, Hangzhou, Zhejiang Province 310053 China; 7 Customer-oriented Wellbeing and Health Hub, Metropolia University of Applied Sciences, Myllypurontie 1, Helsinki, 00920 Finland; 8 Department of Neuroscience and Biomedical Engineering, Aalto University, Otakaari 3, Espoo, 02150 Finland; 9 Finnish National Institute for Health and Welfare, Mannerheimintie 166, Helsinki, 00300 Finland

**Keywords:** Patient-centred measures, Patient-reported measures, ICHOM standard set, PCB set, Acceptability, Feasibility, Adaption, Maternity care

## Abstract

**Background:**

Systematically using standard patient-reported measures (PRMs) in clinical routines is trending. The International Consortium for Health Outcomes Measurement (ICHOM) has developed condition-specific standard sets of patient-centred measures, one of which is the Pregnancy and Childbirth Standard (PCB) set, where standard PRMs are included. There is limited knowledge on the use of ICHOM PCB set-included PRMs (ICHOM-PCB-PRMs) in routine care. This study investigates women’s perspectives on the future implementation of standard ICHOM-PCB-PRMs in routine maternity care in Finland.

**Methods:**

Semi-structured interviews were conducted. Pregnant and postpartum women were asked to evaluate each ICHOM-PCB-PRM in several dimensions, e.g., importance and quality of questions, and to provide their views on future implementation in terms of benefits, difficulties, and practices. With the predefined topics and themes, deductive analysis was applied. Ethical committee approval (HUS 220/880/2015) and research permissions were obtained.

**Results:**

22 women participated. Participants felt that most of the ICHOM-PCB-PRMs were important, relevant, understandable, and appropriately designed, and agreed that some changes in ICHOM-PCB-PRMs were needed, e.g., adding other important measures, changing the wording, and adding open-ended questions. Women would be hesitant to answer questions honestly if follow-up actions were unclear. Most “outcome” measures could be asked repeatedly as maternal health status changes over time, and “experience” measures could be asked separately for different service providers. Disagreements regarding data collection at birth were observed. PRMs were regarded as a way for women to express their thoughts and feelings. Our participants were concerned about the possible consequences of negatively answering the PREMs questions and the availability of follow-up care. Participants expected that they could answer short and easy questions digitally before appointments, and that instructions and follow-up actions based on their answers should be available.

**Conclusion:**

ICHOM-PCB-PRMs could be applicable in Finnish maternity care, but some modifications may be required. Careful consideration is needed regarding how and when PRMs questions are asked for eliciting more accurate and honest answers and minimizing women feeling judged, embarrassed, or offended. Follow-ups should be available according to women’s responses and needs. This study provides insights on the adoption and implementation of standard PRMs in routine maternity care.

**Supplementary Information:**

The online version contains supplementary material available at 10.1186/s12913-023-09818-5.

## Introduction

Systematically using standard patient-centred measures in clinical routines could help professionals to consistently monitor healthcare quality, facilitate patient-centred care, and increase the feasibility of benchmarking for quality improvement [1–3]. The International Consortium for Health Outcomes Measurement (ICHOM) has brought together groups of professionals along with patient representatives to develop condition-specific standard sets of patient-centred outcomes [4], one of which is the pregnancy and childbirth (PCB) set for the childbearing population [5, 6]. The PCB set aims to empower women as active participants in their care, and help professionals make better decisions [5].

The essence of ICHOM standard sets is patient-reported measures (PRMs), which are used to reveal the outcomes (PROMs) and experiences (PREMs) of health services as described by patients; and have been considered important in developing patient-centred and value-based health care [7, 8]. Standard PRMs defined in the ICHOM PCB set (ICHOM-PCB-PRMs) covers multiple issues, including health related quality of life, pain with intercourse, confidence with role as a mother, mother’s attachment to infant (i.e., mother-infant attachment defined in ICHOM PCB standard), breastfeeding, postpartum depression, satisfaction with care, confidence in healthcare providers and birth experience [5, 6]. Additional file 1 presents the details of the ICHOM-PCB-PRMs. Currently, PRMs have been used for research and clinical practices across different medical areas. Most of the published PRMs implementation studies have centred on certain medical areas, such as oncology [1], chronic diseases like epilepsy [9], and mental health [10]. Research on and practices of systematically using PRMs clinically as part of routine care are quite limited in this field.

A few studies, with limited research contexts, investigating the applicability and feasibility of the ICHOM PCB set that includes a series of PRMs in the pregnancy and childbirth pathways have been published recently. There are some studies from the Netherlands, Australia, and Kenya, among which two pre-implementation studies explored the feasibility, barriers, and enablers of using the set with both clinical and patient-reported measures [11, 12]; and seven post-implementation studies analysed the feasibility of using the PRMs defined in the PCB set [13–19]. Currently, limited evidence has tentatively suggested the feasibility and acceptability of applying the ICHOM PCB set. However, knowledge of the adaption of the PCB set, especially the included PRMs, is still lacking for local routine maternity care.

Currently published knowledge and experience are certainly not sufficient for widely using the ICHOM-PCB-PRMs in Finland, which, as many other countries, does not yet systematically collect and use PRMs data in public maternity care. Our previous effort only explored professionals’ views towards the introduction of ICHOM-PCB-PRMs into public maternity care pathway [20]. There is still a lack of perspectives from women who are the centre of care. This pre-implementation study aimed to explore the acceptability and feasibility of ICHOM-PCB-PRMs in Finnish public maternity care from women’s perspectives, and suggested solutions for adaptation. Our previous publication provided the basic information about Finnish healthcare and described the general process of Finnish public maternity care [20]. Briefly, over 99.5% of all pregnant mothers in Finland seek care from public maternity service perinatal care, which is free of charge. Pregnant women obtain perinatal care from Neuvola (community-based maternity and child health clinics led by public health nurses and medical doctors) and get special care and delivery services from delivery units of district hospitals, where midwives are the main service providers for uncomplicated childbirth [20–22]. There are nine antenatal visits and three postpartum maternal check-ups offered to normal pregnancies, ending with an extensive doctor’s check-up at Neuvola between two and four months after childbirth [20].

## Materials and methods

### Study design

In this pre-implementation qualitative study [23, 24], semi-structured interviews were conducted with pregnant and postpartum women to explore the potential of using ICHOM-PCB-PRMs in Finnish maternity care. Since our previous study found that race/ethnicity was not allowed to be asked from patients for medical purposes in Finland [20], the question was removed for this study. Questions about obstetric history were also omitted as service providers could obtain this information from the patient information systems. As in Finland it is not recommended to feed babies under the age of 6 months with water, “water” was removed from the measure of *success with breastfeeding*. To compare viewpoints between professionals and women, a similar interview structure and protocol was applied in this study [20]. During the interviews, participants were first asked to evaluate each ICHOM-PCB-PRM in terms of importance and relevance, time points of data collection, quality of questions, and willingness to answer; and then to provide their views regarding future implementation in terms of benefits or motivations, possible difficulties or risks, and preferred practices or conditions. This study was approved by the Helsinki University Hospital (HUS) Ethical Committee (number: HUS 220/880/2015).

### Participant recruitment

This empirical study was conducted in Helsinki and Uusimaa Health District (Finnish name: Helsingin ja Uudenmaan Sairaanhoitopiiri, HUS), the biggest health district taking over 35% of all deliveries in Finland [25]. In May of 2021, we started to recruit pregnant and postpartum women from different service sites, including Neuvola, HUS prenatal screening unit, and a family coaching session organized by the city of Helsinki. We employed a purposive sampling strategy to create a sample with Finnish background but having heterogeneous characteristics to ensure that the sample was evenly distributed in terms of pregnancy and postpartum stage, gravidity, and parity. Analysis was performed immediately after each interview, and we continued to recruit participants and conduct the interviews until data saturation was reached, i.e. the additional data made little change in analytic patterns and themes [26]. The last interview was conducted on 5th of November, 2021. Nurses and midwives working in the recruitment sites were introduced to this study ahead of time in meetings that were organized by the research team. They got a document of describing the research, a file about recruitment protocol and process as well as copies of informed consent form that would be presented to women. Nurses and midwives prepared themselves with these materials before recruiting women. Participation was voluntary. At the recruitment sites, women were informed by nurses and midwives about the purpose and protocol of the interviews. After counselling, women who were willing to participate were asked to provide basic background information and sign an informed consent document with questions on their demographic background. After signing the consent forms, women sent their signed forms to researchers at HUS with prepaid envelopes via an internal mail system. They were later contacted by one of our researchers (KV), who arranged and conducted interviews with the participants. After completing the interview, participants could obtain a shopping gift card worth €20.

### Data collection and analysis

We commenced the interviews in May 2021 after we got ethical approvals and research permissions and ended in November 2021. The topic guide and a structure of data analysis with predefined themes were developed for this study by reviewing other relevant studies [11, 12, 16, 17, 19] and with the knowledge and experience obtained from our previous study that interviewed Finnish local professionals [20]. Researchers AC and KV did a desk study and established a pool of topics and themes for the research group to discuss and decide which topics and themes might be relevant and important in Finnish context. Additional file 2 presents the structure of data collection and analysis with predefined topics and themes (Please see additional file 2). In the first topic evaluation on ICHOM-PCB-PRMs, women were asked to review and evaluate each measure with four predefined themes: *importance and relevance, time points of data collection, quality of questions, and willingness to answer*; The second topic, women’ views on the future implementation of PRMs, were investigated with three themes: *expected benefits of implementing PRMs or motivations to respond to PRMs questions, possible difficulties or risks, and preferred practices or conditions.* Additional file 3 provides the interview protocol and main questions that were translated into Finnish. The whole interview consisted of three parts: (1) women’s expectations and experiences in the process of pregnancy and childbirth, (2) evaluation on ICHOM-PCB-PRMs, and (3) views on the future implementation of PRMs in Finnish maternity care routine. This paper presents the results of interview part 2 (evaluation on the measures) and interview part 3 (views on the future implementation of PRMs in Finnish maternity care routine) designed to explore women’s perspectives and views on ICHOM-PCB-PRMs and the future implementation. Interview questions were well structured, but women were provided space to freely bring up comments and other topics.

Women read the PRMs during interviews and gave their views and opinions on each PRM according to interview questions. Considering the COVID-19 pandemic situation, we organized interviews via phone or Microsoft Teams. The list of ICHOM-PCB-PRMs was send to women before interview, so that they could see and read the measures during interview, but they were not asked to read and respond to measures prior to interview. The interviewer also read the measures to women during interviews and asked women’s views and opinions on the measures. KV, being proficient in both Finnish and English, conducted interviews in Finnish, tape recorded the interviews, listened to the records, and worked together with AC in transcribing the conversations sentence by sentence from Finnish to English. All English transcripts were imported into Atlas.ti qualitative data analysis software (V.22) for analysis.

With predefined structure and themes, deductive content analysis [27] was employed. In the analysis of women’s evaluation of ICHOM-PCB-PRMs, AC and KV read all comments given by participants under each evaluation dimension (i.e., theme) - *importance and relevance, time points of data collection, quality of questions*, and *willingness to answer*, combined the same and similar comments, and counted the number of participants who mentioned them. For each theme, key comments mentioned by at least five (22.73%) of the participants were reported. Missed measures reported by participants were listed. For analysing the women’s views on implementing PRMs in routine maternity care, we used a deductive approach and organized participants’ answers into three predefined themes- *expected benefits of implementing PRMs or motivations to respond to PRMs questions, possible difficulties or risks*, and *preferred practices or conditions*. For each theme, we combined the same and similar comments and counted the number of women who mentioned them. In the process of analysis, KV and AC analysed the data independently with the agreed structure and themes, compared the intermediate results during the process, and had discussions to reach a consensus.

## Results

### Basic characteristics of participants

We interviewed 22 participants, of whom approximately half were pregnant at the time of the interview, and half had experienced their first pregnancy. Most participants were generally healthy from their own perspectives. Table 1 summarizes the participants’ basic characteristics. Each interview with three parts lasted from 1 to 1.5 h. This paper reported the results of interview part 2 (women’s assessment on ICHOM-PCB-PRMs) and interview part 3 (women’s views on future implementation of PRMs).

**Table 1:** Basic characteristics of participants

Characteristics of participants	Overall (N = 22) (%)
Age (mean, minimum-maximum and SD)	32.05, 24–41, 4.70
Age ≥ 35	8
Age < 35	14
Education (n) (%)	
1. Basic education or less	0
2. Secondary education or vocational qualifications	3 (13.64)
3. Bachelor level	5 (22.73)
4. Master level	13 (59.09)
5. Licentiate or doctor’s degree	1 (4.55)
Employment status (n) (%)	
1. Student	2 (9.09)
2. Employed	19 (86.36)
3. Unemployed	1 (4.55)
Pregnancy/Postpartum stage (on the date of interview)	
1. First trimester and second trimester	6 (27.27)
2. Third trimester	6 (27.27)
3. One to three months postpartum	6 (27.27)
4. After three months postpartum	4 (18.18)
Gravidity, including current pregnancy (n) (%)	
1. One	11 (50.00)
2. More than one	11 (50.00)
Parity (n) (%)	
1. None	9 (40.91)
2. One	8 (36.36)
3. More than one	5 (22.73)
Health status from women’s own perspectives (n) (%)	
1. Generally healthy	15 (68.18)
2. Chronic diseases, gestation-related or birth-related health problems or other health problems	7 (31.82)

### Assessment on ICHOM-PCB-PRMs

#### Importance and relevance

In general, participants felt that ICHOM-PCB-PRMs were important and relevant to Finnish maternity care. Women emphasized the importance of questions about social networks, support, and mental health. Over two-thirds of the participants pointed out that education might not be important for getting equal and high-quality maternity care from public service and suggested the removal of this question. *Incontinence* was the measure that many participants found unfamiliar, since they had little experience or knowledge about this medical problem. Over 20% participants reported concerns on the relevance of *pain with intercourse*, *success with breastfeeding*, *confidence as an active participant in healthcare decisions* and the ‘hygiene’ question in *birth experience*. More than half of the participants pointed out that some measures were missing from the list of ICHOM-PCB-PRMs, including preparation for birth, caesarean section process and experience, burden of childcare, and family situation. Table 2 shows the participants’ key views on the importance and relevance of each ICHOM-PCB-PRM and missed measures.

**Table 2:** Importance and relevance of ICHOM-PCB-PRMs for Finnish maternity care and missed measures

Patient reported measures	Key points with number of participants and percentage	Sample quotations Age, parity, gestation weeks/postpartum months
Patient reported case-mix variables	These measures are important (n = 22, 100%). Education was not found important (n = 15, 68.18%). The question about social network and supports was found to be especially important by half (n = 11, 50.00%).	*I would say that these are useful for Neuvola to know. To know the background of the mother and the family. Makes it easier to target the service. (31, G2P1, H32 + 2)* *Education is also a question that “why do you ask” in my point of view. (29, G2P0, H29 + 2)* *The question about social network and supports should be asked repeatedly during pregnancy. (30, G1P0, H28 + 1)*
PROMs Health related quality of life	These measures are important (n = 22, 100%). Mental health questions were considered especially important (n = 12, 54.55%).	*I think these are really good and relevant questions. When I had a burnout and depression, these were the kind of issues I handled. (29, G2P0, H29 + 2)* *Giving birth is a huge change of life. And by asking these you get the feeling of being cared for, and that mental wellbeing is important. (40, G1P1, 5 weeks postpartum)*
PROMs Incontinence	These measures are important (n = 20, 90.91%). Relevant to ask so that one could get help if needed (n = 6, 27.73%). Measures were felt important but not familiar because of little experience or knowledge about the concept (n = 9, 40.91%).	*I have never seen these kinds of questions asked. I would say yes. Because these are embarrassing issues that one maybe doesn’t want to talk about if not asked. These are good. (25, G2P1, H32 + 2)* *But these went to a really detailed level. It would be important that somebody who really goes through these should get help. And women should be given support and help even if the problem is not so bad, at a lower threshold. (32, G3P3, 1 month postpartum)* *I have never been asked about these at Neuvola. (25, G1P0, H30 + 5)*
PROMs Pain with intercourse	These measures are important (n = 17, 77.27%), especially for the relationship. These were not relevant if one didn’t have sex at the time (n = 5, 22.73%).	*I suppose this influences the partnership a lot. (31, G2P1, H32 + 2)* *A couple’s sexual behaviour is important and may cause conflicts, and is also a sensitive issue. (25, G2P1, H32 + 2)* *Is there really a need to ask these questions during pregnancy? Unless there are some problems with it. Maybe this should be asked after birth, in Neuvola, after first follow-up (2–4 months postpartum), because many don’t have sex so soon after birth. (29, G1P1, 2 months postpartum)*
PROMs Confidence with role as a mother	All thought these measurements were important (n = 22, 100%).	*Yes, this is important. And if one doesn’t have experience yet, then it should be asked if she is nervous about it. (30, G2P2, 4 months postpartum)* *Absolutely yes. I didn’t have personal experience with kids before I had my own. So, I have had to think and process quite a lot by myself and learn more. So, it is really good to ask. (36, G2P1, 2 months postpartum)*
PROMs Mother-infant attachment	Questions are important (n = 18, 81.82%).	*All kinds of emotions are normal. And someone might get scared if she has some negative feelings. Then everything is not nice. And there might be postpartum depression problems. (28, G1P0, H24 + 4)* *For me, with this third child I have more positive feelings compared to the first one. These questions might raise thoughts during pregnancy. (32, G3P3, 1 month postpartum)*
PROMs Maternal confidence with breastfeeding	These questions are important (n = 17, 77.27%).	*Maybe it is difficult to think of those during pregnancy. There maybe should be a choice that “I don’t know yet,” and that might start a conversation in Neuvola. It felt difficult in Neuvola when I was asked this, I said that I really don’t know yet. (31, G2P1, H32 + 2)* *This is interesting. Because the breastfeeding theme is important. (32, G2P2, 6 months postpartum)*
PROMs Success with breastfeeding	These questions were important (n = 19, 86.36%). Relevance depended on the situation (n = 6, 27.73%).	*Yes, it would be important to ask how this is going. I think this should be talked about to find out if there are any problems, and what are the mothers’ feelings. I have heard that someone has exhausted herself with this issue and felt like a bad mum when she didn’t succeed*. (*25, G1P0, H30 + 5)* *What is the purpose? I would want to add a question in the future that if breastfeeding didn’t succeed at first, would you want to have another try later? (32, G3P3, 1 month postpartum)*
PROMs Postpartum depression	These are important and relevant questions (n = 22, 100%).	*Yes, absolutely. These cases need to be found. And this might help those people who wouldn’t want to talk about problems otherwise. (30, G2P1, H24 + 4)*
PREMs Satisfaction with the result of care	These are important and relevant questions (n = 22, 100%).	*I was hoping for this kind of question. (39, G1P0, H27 + 2)*
PREMs Confidence as an active participant in healthcare decisions	These questions are important and relevant. Also, these raise some concerns (n = 21, 90.91%). Choices and decisions are not relevant in some contexts (n = 5, 22.75%).	*Yes. So that one can find out how to do better in the future if the confidence is not so good. This is also an important issue so that service can be improved. (31, G2P1, H32 + 2)* *This question about being an active participant in healthcare decisions really aroused feelings in me. The decisions and actions of professionals are questioned. But I feel that nowadays people think that they have a right to make the decisions about everything in healthcare. (36, G2P1, 2 months postpartum)* *I don’t really know what the healthcare decisions are during pregnancy. I have just gone with the flow myself. (30, G1P0, H28 + 1)*
PREMs Confidence in healthcare providers	These questions are important and relevant (n = 18, 81.82%).	*Kind of yes, important question, but this too could be difficult to answer if the one who is asking is the one you are disappointed with. (35, G1P0, H20 + 6)* *Yes, I think so. Especially if the delivery was difficult and something went wrong. So, there might be FOB the next time. This subject should be talked through already at this stage, and not just in the next possible pregnancy. (25, G1P0, H30 + 5)*
PREMs Birth experience	These questions are important and relevant (n = 22, 100%). Question about the delivery rooms being clean and hygienic was found not so important (n = 5, 22.73%). Questions are not relevant for Cesarean section (n = 5, 22.7%).	*Yes. These concern satisfaction. I think that almost everything can be improved. That’s why they are important questions. (24, G1P0, H30 + 3)* *Maybe the last one about the cleaning of the delivery room. Does that need to be asked? I think that should be obvious. (41, G4P0, H19 + 6)* *What about caesarean section? (41, G4P0, H19 + 6)*

**Missed measures**: 12 participants (54.55%) pointed out that some measures were missing from the list of ICHOM-PCB-PRMs.

• Eight participants (36.36%) suggested to include measures about **preparation for birth**, such as confidence in birth, fear of childbirth, knowledge about birth, and birth wishes.

• Three participants (13.64%) pointed out that measurement on birth process and experience should include **specific questions for the case of caesarean section.**

• Three participants (13.64%) thought **workload and experienced burden of taking care of babies** should be measured.

• Two participants (9.09%) said that questions about **family situation**, such as financial situation and family relationship, should be asked.

#### Quality

Table 3 displays the assessments of the quality of ICHOM-PCB-PRMs in terms of comprehensiveness, appropriateness of options and scales, and difficulties in responding. According to the participants’ feedback, most measures and questions were understandable. Some reported that questions in the measurement of *confidence in breastfeeding* were not understandable; it was especially difficult for first-time mothers who lacked experience in understanding those questions. To some extent, these questions were prejudiced against non-breastfeeding mothers, and imposed pressure on the women who failed to breastfeed.

**Table 3:** Quality of ICHOM-PCB-PRMs questions in terms of comprehensiveness, appropriateness of options and scales, and perceived difficulties in responding to questions

Patient reported measures	Key points with number of participants and percentage	Sample quotations Age, parity, gestation weeks/postpartum months
Patient reported case-mix variables	These measures are understandable (n = 22, 100%). These options are appropriate (n = 19, 86.36%). These questions are not difficult to answer (n = 17, 77.27%). The question about social network might be difficult; it should be asked differently (n = 8, 36.36%).	*Questions are understandable and easy to answer. (29, G2P0, H29 + 2)* *Question about social network is a bit difficult, like how to quantify those people who can help you? But maybe it is better to really think about those persons from whom I would really ask for help? We have that neighbour there, but would I really count her in or not, then not. So, this is a good question. This is an old question, but it’s asked differently. Not just ask “Do you feel you have a sufficient social network?” (25, G2P1, H32 + 2)*
PROMs Health related quality of life	These measures are understandable (n = 18, 81.82%). These options are appropriate (n = 19, 86.36%). Some questions are difficult for the women to answer (n = 12, 54.55%). Open answers should be allowed (n = 5, 22.73%).	*Yes, they are understandable. I think the term “in general” is a bit difficult. (29, G2P0, H29 + 2)* *Yes, it is better than just yes/no answers. (35, G1P0, H20 + 6)* *Might be difficult if you have mood changes .It would help if these were asked repeatedly (32, G3P3, 1month postpartum)* *Maybe there should be a free speech part (39, G1P0, H27 + 2)*
PROMs Incontinence	These measures are understandable (n = 22, 100%). These options are appropriate (n = 22, 100%). These questions are not difficult to answer (n = 18, 81.82%). These might be a bit embarrassing (n = 7, 31.82%).	*I have heard that these are embarrassing issues to talk about that one might not want to or dare to speak of. (30, G1P0, H28 + 1)*
PROMs Pain with intercourse	These measures are understandable (n = 22,100%). These options are appropriate (n = 18, 81.82%). These questions are not difficult to answer (n = 17, 77.27%). It is difficult to answer if women haven’t had sex yet. (n = 5, 22.73%).	*Question is understandable but I would like it to be more specific. I was thinking that should it be more specific, a question like what kind of pain, or is the pain in the stomach (pressure) or in the vagina. (29, G2P0, H29 + 2)* *Limit of 30 days might be too short. Not all are so active during pregnancy or after birth? (32, G3P2, 2 months postpartum)*
PROMs Confidence with role as a mother	These measurements are understandable (n = 22, 100%). These options are appropriate (n = 18, 81.82%). These questions are not difficult to answer (n = 15, 68.18%). Women may feel pressed if they are asked or the answers are negative (n = 5, 22.73%).	*This might feel like a bit of a sensitive question. One might think that she is thought to be a bad mother because this is asked. Or if she is unconfident (40, G1P1, 5 weeks postpartum)*
PROMs Mother-infant attachment	These measurements are understandable (n = 21, 95.45%). These options are appropriate (n = 15, 68.18%). Words used to describe feelings are too strong and negative. (n = 5, 22.73%). These questions are not difficult to answer (n = 13, 59.09%). It is difficult to answer because one doesn’t know what would happen if one answered negatively (n = 7, 31.82%).	*These are very strong words. Maybe it would be better to ask what kind of adjectives one would use to describe the baby? These are too strong words. I don’t think anyone would feel aggressive or disappointed towards the baby but maybe with some situations. (40, G1P1, 5 weeks postpartum)* *But does one dare to answer honestly if one had some negative thoughts? And one could think that what could happen if you have negative thoughts. Like in Facebook conversations some are afraid of social workers if they told negative thoughts*. *(32, G3P3, 1 month postpartum)*
PROMs Maternal confidence with breastfeeding	These measures are understandable (n = 16, 72.73%). Some questions are difficult to understand and need to be modified (n = 7, 31.82%). These options are appropriate (n = 15, 68.18%). More options are needed (n = 8, 36.36%). These questions are difficult to answer (n = 11, 50%). Difficulties could be found if the participant didn’t have experience yet (n = 11, 50. %).	*Some questions are a little bit strange, like what does it mean “I can always continue to breastfeed my baby for every feeding”? Maybe that one keeps on breastfeeding and not give formula? Is the question about “maternal confidence with breastfeeding” a bit prejudiced? So, is it a failure if one doesn’t breastfeed? I wouldn’t say anything about succeeding. (25, G2P1, H32 + 2)* *There maybe should be a choice that “I don’t know yet” and that might start a conversation in Neuvola. (31, G2P1, H32 + 2)* *It is hard to answer these questions without experience. (24, G1P0, H30 + 3)*
PROMs Success with breastfeeding	These measures are understandable (n = 21, 95.45%). These options are appropriate (n = 19, 86.36%). These questions are not difficult to answer (n = 22, 100%)	*Yes, it is understandable. (40, G1P1, 1 month postpartum)*
PROMs Postpartum depression	These measures are understandable (n = 20, 90.91%). These options are appropriate (n = 17, 77.27%). These questions are not difficult to answer (n = 14, 63.64%). It might be difficult to give accurate and honest answers (n = 9, 40.91%).	*Maybe here is a problem with the scaling. (37, G2P2, 7 months postpartum)* *How brave you would be to answer this honestly? (30, G2P1, H25 + 4)*
PREMs Satisfaction with the result of care	These measurements are understandable (n = 19, 86.36%). These options are appropriate (n = 16, 72.73%). These questions are not difficult to answer (n = 12, 54.55%). Difficulties were found if the one is not satisfied with the one who is asking (n = 14, 63.64%). Questions should be asked separately for different providers involved in the care process (n = 11, 50.00%).	*There might be a problem with this measure. Who will ask this and how will these issues be handled? Do you want to tell if you are really disappointed in your car? Will the answers go to your own nurse in Neuvola? (29, G2P0, H29 + 2)* *I think the evaluation of care given at Neuvola, the screening unit, the hospital and my home after birth should be separated. (39, G1P0, H27 + 2)*
PREMs Confidence as an active participant in healthcare decisions	These measures are understandable (n = 19, 86.36%). These options are appropriate (n = 21, 95.45%). Open answers should be allowed (n = 5, 22.73%). These questions are not difficult to answer (n = 17, 77.27%). It is difficult to answer the questions because different providers are involved. Questions should be asked separately for different providers (n = 9, 40.91%). Giving direst negative feedback might be difficult (n = 5, 22.73%).	*These might be hard to answer. I would prefer to answer open-ended questions than these. (35, G1P0, H20 + 6)* *Who is asking and how these would influence the care in future? (29, G2P0, H29 + 2)* *It could be difficult to give negative feedback straight to those persons working there. (25, G2P1, H32 + 2)* *I think the evaluation be separated for different providers. (39, G1P0, H27 + 2)*
PREMs Confidence in healthcare providers	These measurements are understandable (n = 22, 100%). These options are appropriate (n = 17, 77.27%). Open answers should be allowed (n = 5, 22.73%). These questions are not difficult to answer (n = 14, 63.64%). It is difficult to answer question because different providers are involved (n = 5, 22.73%).	*Here too should be an “open text” part. Opportunity is required to tell what went wrong. (30, G2P1, H25 + 4)* *There are so many different people in healthcare. So, you might feel confident for some and not so confident for others. This could be difficult to answer. Could be asked in the hospital after birth. But this should be a more open-ended question. (35, G2P2, 7 months postpartum)*
PREMs Birth experience	These questions are understandable (n = 22, 100%). These options are appropriate (n = 19, 86.36%). These questions are not difficult to answer (n = 18, 81.82%).	*Well, hard for me to say now because I don’t have the experience. But these are quite concrete so I suppose these would not be difficult to answer. If someone has had a very traumatic delivery, then it could be difficult to handle these questions. (26, G1P0, H21 + 4)*

The design of the questions in terms of the scales used and available options was appropriate and acceptable in general. However, over one-third of the participants criticized the scale used for social networks and support. Some reported that words of feeling used in the measurement of *mother-infant attachment* were too strong and negative. Some suggested that questions of *confidence with breastfeeding* could have more options, such as “I don’t know” and allow qualitative responses. The possibility of answering in one’s own words to questions of *health-related quality of life*, *confidence in healthcare providers*, and *confidence as an active participant in healthcare decisions* was requested by some.

Over one-third of the participants mentioned that the issue of social networks and support might be difficult to answer because quantifying this was problematic. Approximately one-third pointed out that it might be embarrassing to respond to *incontinence*. Some were worried that the questions of *pain with intercourse* would be difficult to answer if the woman hadn’t had sex yet after giving birth. Several participants felt hesitant to give honest answers to *confidence in their role as a mother*, *mother-infant attachment*, and *postpartum depression* if their answers would have been negative, and were concerned about the possible consequences. The participants pointed out that it was difficult to provide negative feedback in a non-anonymous questionnaire with PREMs. Many interviewees also felt that it was difficult to answer *satisfaction with the result of care*, *confidence as an active participant in healthcare decisions*, and *confidence in healthcare providers*, because in Finland, different care providers were involved in maternity care.

#### Willingness to answer

Table 4 summarizes the participants’ willingness to answer the ICHOM-PCB-PRMs questions. Most participants expressed a high willingness to answer the PRMs. Some said that answering *incontinence* questions would depend on the availability of help, connection with the person who would ask, and privacy. Over one-third of participants said answering *mother-infant attachment* questions would depend on the availability of help, the purpose of the questions, and the possible consequences of negative answers. The same number of participants said answering questions of *satisfaction with the result of care* depends on who would ask these questions and what would be the consequences of answering the questions.

**Table 4:** Willingness to answer ICHOM-PCB-PRMs questions

Patient reported measures	Key points with number of participants and percentage	Sample quotations Age, parity, gestation weeks/postpartum months
Patient reported case-mix variables	These measurements are answered willingly (n = 22, 100%).	*I know that many women don’t like to tell their height and weight. Those are quite delicate issues. I wouldn’t mind talking about those, but I know some might. Some women don’t even want to get their weight measured in Neuvola. (29, G2P0, H29 + 2)*
PROMs Health related quality of life	These measurements are answered willingly (n = 20, 90.91%).	*I would be happy to answer these questions. (29, G2P0, H29 + 2)*
PROMs Incontinence	These measurements are answered willingly (n = 17, 77.27%). Willingness depends on some factors, including the availability of help, the connection with the person who is asking, and privacy (n = 6, 27.73%).	*If it’s about my wellbeing, then yes. And I would get some help after answering. (29, G2P0, H29 + 2)* *If I would get some help after answering, I am willing to answer the questions. (32, G3P3, 1 month postpartum)*
PROMs Pain with intercourse	These measurements are answered willingly (n = 19, 86.36%).	*Not maybe happily if I had problems, but yes, I would answer. (35, G1P0, H20 + 6)* *This might be easier to answer in “Maisa” (an online patient portal). This kind of personal question would be easier to answer like that and then discussed in Neuvola if needed. (30, G2P1, H25 + 4)*
PROMs Confidence with role as a mother	These measurements are answered willingly (n = 18, 81.82%).	*I am willing to answer these questions. But formulation of the question should be inclusive. For me the question should be “Confident with role as a parent,” not “a mother”.” (41, G4P1, H19 + 6)*
PROMs Mother-infant attachment	These measurements are answered willingly (n = 16, 72.73%). Willingness depends on the availability of help, the purpose of questions, and possible consequences of negative answers (n = 7, 31.82%).	*I would like to answer these questions and I feel I need help (24, G1P0, H30 + 3)* *I wonder if I would dare to answer honestly if I had negative thoughts. (30, G2P1, H25 + 4)* *Maybe here too should be a brief introduction that it’s natural to feel many things. Because some might feel like a bad mother if they choose some negative things. (35, G1P0, H20 + 6)*
PROMs Maternal confidence with breastfeeding	These measurements are answered willingly (n = 19, 86.36%).	*Yes, then one could find out how this is going. (31, G2P1, H32 + 2)*
PROMs Success with breastfeeding	These measurements are answered willingly (n = 20, 90.91%).	*I would like to answer these questions. But why is this asked? Is it that breastmilk is good, but formula is not good for your baby? So, what then? Would there be advise? What will happen if I say that I have been giving formula? What is the amount of milk? (39, G1P0, H27 + 2)*
PROMs Postpartum depression	These measurements are answered willingly (n = 20, 90.91%).	*Yes, I will answer these questions if everything was well. But maybe not if I had some problems. (30, G2P2, 4 months postpartum)*
PREMs Satisfaction with the result of care	These measurements are answered willingly (n = 19, 86.36%). There are concerns about who is asking these (n = 7, 31.82%).	*Kind of yes, but then I would wonder about its effect. Like if I weren’t very pleased with some place and they get to see the answers, so how will the answers be handled? There is a fear of how it would affect future treatment (41, G4P0, H19 + 6)* *It will depend on who is asking. If you want to give feedback, you have to have the courage to say it. It’s like a “double-edged sword” if it’s asked by your nurse in Neuvola and you are not too happy during pregnancy. If the chemistry doesn’t work. (37, G2P2, 7 months postpartum)*
PREMs Confidence as an active participant in healthcare decisions	These measurements are answered willingly (n = 16, 72.73%).	*Kind of yes, but then I would wonder about its effect. (41, G4P0, H19 + 6)* *Like before, if the one who was asking these would be the one that I was not happy with, I don’t know if I would dare to answer. (36, G1P0, H21 + 4)*
PREMs Confidence in healthcare providers	These measurements are answered willingly (n = 18, 81.82%).	*Yes, I am willing to. But if you are afraid that these might influence your treatment in the future, then these would be difficult to answer. Anonymous would make it easier. It would be good that one could answer anonymously. (30, G1P0, H28 + 1)*
PREMs Birth experience	These measurements are answered willingly (n = 22, 100%).	*Yes, I am willing to. But I feel this is not so much for mothers who underwent a Sect. (32, G4P1, 2 months postpartum)*

#### Timing of answering

Many participants agreed that most measures, including *health-related quality of life*, *pain with intercourse*, *confidence with the role as a mother*, *mother-infant attachment, success with breastfeeding*, *postpartum depression*, *satisfaction with the result of care*, *confidence as an active participant in healthcare decisions*, and *confidence in healthcare providers*, as well as social networks and support, should be asked repeatedly during the care pathway, as the status might change. There was disagreement regarding the appropriateness of asking *about confidence in breastfeeding* before birth. Some participants pointed out that some measures, such as *pain with intercourse* and *postpartum depression*, should not be asked too early after birth. Table 5 shows the participants’ views on the time points of answering the ICHOM-PCB-PRM questions.

**Table 5:** Preferred time points of answering ICHOM-PCB-PRMs questions

Patient reported measures	First trimester	Second trimester	Third trimester	Soon or early after birth	One month postpartum	Three months postpartum	Six months postpartum	Key comments from women (more than five women mentioning)
Patient reported case-mix variables	n = 21, 95.45%	None	None	None	None			Some questions, e.g., social support, could be asked repeatedly during pregnancy (n = 11, 50.00%).
PROMs Health related quality of life	n = 9, 40.91%	n = 6, 27.73%	n = 8, 36.36%	None	After birth, but not immediately, n = 19, 86.36.%			This should be asked repeatedly during pregnancy and after birth (n = 14,63.64%).
PROMs Incontinence	n = 2, 9.09%	None	n = 7, 31.82%	None	After birth, but not immediately, n = 18, 81.82%			None
PROMs Pain with intercourse	During pregnancy, n = 15, 68.18% I trimester, n = 5, 22.73% II trimester, n = 3, 13.64% III trimester, n = 2, 9.09%			None	After birth, n = 15, 68.18% Two to four months after birth during follow-up visit, n = 6, 27.73%			This should not be asked too early after birth (n = 6, 27.73%) This should be asked repeatedly during pregnancy and after birth (n = 7, 31.82%).
PROMs Confidence with role as a mother	n = 2, 9.09%	n = 4, 18.18%	n = 12, 54.55%	n = 8, 36.36%	After birth but not too early, n = 12, 54.55%			This should be asked repeatedly during pregnancy and after birth (n = 5, 22.73%).
PROMs Mother-infant attachment	During pregnancy, n = 5, 22.73% I trimester, n = 1, 4.55% II trimester, n = 1, 4.55% III trimester, n = 3, 13.64%			n = 8, 36.36%	After birth, n = 15, 68.18% One to two months postpartum, n = 7, 31.82%			This should be asked repeatedly after birth (n = 6, 27.73%).
PROMs Maternal confidence with breastfeeding	During pregnancy, n = 5, 22.73% II trimester, n = 2, 9.09% III trimester, n = 6, 27.73%			n = 9, 40.91%	After birth, n = 16, 72.73%			This should not be asked during pregnancy (n = 5, 22.73%).
PROMs Success with breastfeeding	None			n = 7, 31.82%	After birth, n = 11, 50.00% One month postpartum, n = 6, 27.73% Three to six months postpartum, n = 3, 13.64%			This should be asked repeatedly after birth (n = 7, 31.82%).
PROMs Postpartum depression	During pregnancy, n = 21, 95.45% I trimester, n = 3, 13.64% II trimester, n = 11, 50.00%			None	After birth, n = 21, 95.45% One month postpartum, n = 5, 22.73% Three to six months postpartum, n = 5, 22.73%			This should be asked repeatedly and regularly (n = 12, 54.55%). This should not be asked too early after birth (n = 6, 27.73%).
PREMs Satisfaction with care	During pregnancy, n = 15, 68.18% I trimester, n = 1, 4.55% II trimester, n = 2, 9.09% III trimester, n = 6, 27.73%			n = 9, 40.91%	After birth, n = 15, 68.18% One month postpartum, n = 6, 27.73% Three to six months, n = 4, 18.18%			This should be asked at every stage (n = 7, 31.82%).
PREMs Confidence as an active participant in healthcare decisions	During pregnancy, n = 15, 68.18% I trimester, n = 1, 4.55% III trimester, n = 7, 31.82%			n = 12, 54.55%	After birth, n = 16, 72.73% One to two months postpartum, n = 5, 22.73% Six months postpartum, 1, 4.55%			This should be asked repeatedly (n = 6, 27.73%).
PREMs Confidence in healthcare providers	During pregnancy, n = 13, 59.09% II trimester, n = 2, 9.09%			n = 11, 50.00%	After birth, n = 14, 63.64% One month postpartum, n = 6, 27.73% Two months postpartum, n = 1, 4.55% Few months postpartum, n = 1, 4.55%			This should be asked repeatedly after different stages or events (n = 5, 22.73%).
PREMs Birth experience	None			n = 19, 86.36%	One to two months postpartum, n = 8, 36.36%			This could be asked repeatedly after birth (n = 6, 27.27%).

### Women’s views on the future implementation of PRMs

The participants were asked about their views on the implementation of PRMs. All participants supported the implementation of the PRMs. Table 6 displays the participants’ views on the implementation of PRMs in Finnish maternity care, which were categorized into three primary themes: i.e., expected benefits of implementing PRMs or motivations to respond to PRMs questions, possible difficulties or risks, and preferred practices or conditions.

**Table 6:** Women’s views on the implementation of PRMs in Finnish maternity care

Themes	Key points (over 5)	Sample quotations
Benefits or motivations	Answering PRMs questions is a chance to tell one’s own feelings and be heard (n = 7, 31.82%).	*By answering questions, you are heard and it is easier for the professional too to see what is going on. (39, G1P0, H27 + 2)*
Possible difficulties or risks	Giving negative feedback directly to care provider might be difficult (n = 11, 50.00%).	*I might not want to answer if I had some negative feedback for the care provider who is asking. This could be difficult. But if it was asked, then one could just say “everything is ok” even though they are not thinking so. Then this doesn’t work. (31, G2P1, H32 + 2)*
	It will be difficult to respond to questions if the possible consequences of answering questions and availability of follow-up care are not clear (n = 7, 31.82%).	*I will wonder who will see the answers and how the answer will be addressed. That should be clear beforehand. (32, G2P2, 6 months postpartum)*
	Women’s mental problems, physical problems and difficult circumstances may hinder their responses to the questions (n = 6, 27.27%).	*I would not want to answer the questions if I had some mental issues. (24, G1P0, H30 + 3)*
Preferred practices or conditions	Women should have chances to discuss with professionals and get help based on answers (n = 15, 68.18%).	*I would like to process these questions and answers with some healthcare professionals. Especially if there would be some private questions. (24, G1P0, H30 + 3)*
	Questions should be short, not too many and not asked too often (n = 8, 36.36%).	*Maybe during pregnancy there should be a few short questions only, and more questions if needed. (35, G1P0, H20 + 6)*
	Questions could be answered before appointments (n = 6, 27.27%).	*Maybe one could fill these at home before an appointment at Neuvola. I think these would be nicer to answer at home, so you have time to think about these yourself before talking with healthcare professionals. And then you would go through these with someone. And there would be a way to handle things if needed. Then it would be good to answer in private and with time. (28, G1P0, H24 + 4)*
	It would be convenient to answer digitally (n = 6, 27.27%).	*I would expect questions are in a digital form and answers are filed for different nurses and up to date. (32, G2P2, 6 months postpartum)*
	There should be clear information and instruction on the purpose of questions and possible consequences of answering questions (n = 5, 22.73%).	*A woman should be told that it is important to tell how she is doing physically and mentally. One should not be afraid to answer questions. (31, G1P0, H28 + 1)*

#### Benefits or motivations

Regarding the benefits and motivations, the main benefit of implementing PRMs mentioned by our participants was to give women a feeling of having chances to talk, being heard, and being cared for. Some participants reflected that by answering questions, women could better recognize their health, prepare themselves for visits, and have a better communication with professionals, while professionals can have a better understanding on women’s health status and needs by checking PRMs answers and communicating with women.

#### Possible difficulties or risks

Regarding the difficulties and risks, half of the participants were worried about difficulties in giving negative feedback directly to those providing care; one-third worried that it would be difficult for women to answer questions if the possible consequences of answering questions and the availability of follow-up care are not clear.

#### Preferred practices or conditions

Regarding the preferred practices and conditions, some pointed out that women’s health status and physical circumstances need to be considered, which may affect their ability and willingness to answer the questions, most participants suggested that, after answering the questions, women should be provided with opportunities to discuss with professionals; several participants expressed their preference for short and easy questions; some participants emphasized the convenience of answering the questions digitally and prior to appointments; a few participants stated that there should be clear explanations for why the questions were asked, and women should be provided with clear information and instructions on the possible consequences of answering questions.

## Discussion

This study investigated women’s opinions on ICHOM-PCB-PRMs and their views on future implementation of PRMs in Finnish public maternity care. In this study, quality and acceptability were found to be high in ICHOM-PCB-PRMs. Some issues to routine measurements that were relevant to pregnant and postpartum women in Finland were missing in the list of ICHOM-PCB-PRMs. The willingness of pregnant and postpartum women to answer the ICHOM-PCB-PRM questions was generally high. The potential benefits of implementing PRMs were identify by women, as well as the potential risks and difficulties. Women also provided suggestions on future implementation of PRMs in Finnish public maternity care.

This study could help pre-test ICHOM-PCB-PRMs in the Finnish context and provide insights for adapting and localizing the standard set. According to our results, PRMs listed in the ICHOM-PCB set were generally important and relevant, and the quality of PRMs questions was acceptable, as other studies suggested [13, 19]. But improvements in some measures are required for local use. Table 7 presents the suggested changes for each measure based on women’s views. The ICHOMs PCB set includes five time points of data collection throughout pregnancy and postpartum until six months postpartum. Our study revealed some disagreement between the timing recommended by the ICHOM and what the participants preferred. The main disagreement was related to questions asked at birth or immediately after birth. While ICHOM recommended asking women at birth or just after birth about their confidence and success in breastfeeding and their attachment to the newborn, in our study, less than half of the participants agreed on the importance of asking these questions at birth. More than half of the participants preferred to answer PREMs questions at birth, including *satisfaction with the result of care*, *confidence as an active participant in healthcare decisions*, *confidence in healthcare providers*, and *birth experience*. This is mainly because in Finland, perinatal care and birth care are separate, that is, delivery hospitals offer birth services, and women receive perinatal care from Neuvola. Women would like to give immediate feedback on their service experiences. However, women’s views on the appropriateness of answering questions at birth differed from those of local professionals. According to our previous study, professionals were concerned about women’s health status and medical staff’s workload at birth or just after birth [20]. Thus, questions should be precise and advanced ICT tools should be applied to overcome the possible obstacles in data collection, in that the women can answer questions whenever they feel able to and medical staff can be free from the hypothetically burdensome data collection process. Another conflict we observed between local women’s opinion and the professionals’ view was about the importance of the “education” question. While our professionals agreed that knowing women’s educational level could help to improve communication and provide personalized services [20], our women suggested the removal of the measurement and many of them asked “why this was asked” as they emphasized that one should get equal and high-quality care from public health care system regardless of their education level. As studies have confirmed that women’s educational level could affect women’s health literacy, health behaviors and pregnancy outcomes [28–30], it would be important to motivate Finnish women to provide their education information so that personalized care could be well designed. Providing an explanation of why education level information is important and asked (e.g. for improving health professionals’ understanding on women) and how the information will be used (e.g. supporting health professionals’ to provide women with personalized care) may relief women’s concerns and motivate women to provide their education information.

**Table 7:** Suggestions on the adaptation of ICHOM-PCB-PRMs for local use in Finland

Patient reported measures	Adaptation, rationales, and considerations
Patient reported case-mix variables	Our study suggests that education should be removed from the measurement, as our women believed that one should get equal and high-quality care from public health care system regardless of education level. In contrast, our local professionals reported that education might be important to know for recognizing who may need extra support (Chen et al. 2021).
PROMs Health related quality of life	In addition to predefined options, there should be some space for women to provide open-ended answers or describe their situations in detail. Offering the possibility for women to write free form answers might empower the women more, and help professionals to develop better insights into women’s situations.
PROMs Incontinence	This measure could be removed from regular measurement but asked for details from those who bring up the issue and ask for help.
PROMs Pain with intercourse	This measure could be removed from regular measurement but asked for details from those who bring up the issue and ask for help.
PROMs Mother-infant attachment	Some words like “aggressive” were thought to be too strong and negative and made participants uncomfortable to respond, which should be avoided in questions.
PROMs Maternal confidence with breastfeeding	More explanation may be needed for a first-time mother to answer the questions. Or instead of asking about confidence of breastfeeding that would be difficult for the primiparous to answer, it may be better to ask what kind of information women would need to breastfeed.
PROMs Success with breastfeeding	Non-breastfeeding mothers may feel judged, embarrassed, or offended by the breastfeeding questions. This measure could be removed from regular measurement but asked for details from those who bring up the issue and ask for help.
PREMs Confidence as an active participant in healthcare decisions	This measure could also be removed from regular measurement, as in a normal situation, women may prefer to go with the flow to get health care and do what is recommended by professionals, and information need should be asked. In addition to predefined options, there should be some space for women to provide open-ended answers or describe their situations in detail. Offering the possibility for women to write free form answers might empower the women more, and help professionals to develop better insights into women’s situations.
PREMs Confidence in healthcare providers	This measure could also be removed from regular measurement, as in a normal situation, women may prefer to go with the flow to get health care and do what is recommended by professionals, and information need should be asked. In addition to predefined options, there should be some space for women to provide open-ended answers or describe their situations in detail. Offering the possibility for women to write free form answers might empower the women more and help professionals to develop better insights into women’s situations.
PREMs Birth experience	This is no problems regarding the hygiene of delivery rooms in Finland, so the question about hygiene could be removed from *birth experience*. This is in line with the Finnish maternity care professionals’ view (Chen et al. 2021). However, the BSS-R scale (Birth Satisfaction Scale-Revised) in the ICHOM-PCB set, used to measure *birth experience*, is an international standard measure, so the removal of components from the scale for local use may cause problems in international comparison and benchmarking.

The general attitude towards the implementation of PRMs was positive. Women recognized the value of applying PRMs in routine care. This study suggests practices for implementing PRMs in routine maternity care. Reliable digital tools should be available to efficiently collect self-reported data and allow women to answer questions prior to appointments. According to our previous study based on interviews with professionals, digital tools integrated with electronic medical systems have been used in different health districts of Finland [20]. The new health and social care information system Apotti is used in the capital region, which could help to make ePROMs and ePREMs collection, processing and management possible [20]. However, since different providers are involved in the pathway of public maternity care in Finland, the responsibility and process of data collection, policy of data sharing and integrating, and task division of follow-up actions based on PRMs answers should be clearly defined. A necessary effort is to encourage women to provide honest answers [31–33]. Women should be informed about the purpose of PRMs questionnaires and possible benefits, for example, the collected information will be used to monitor women’s health status, facilitate communication, detect problems, and improve the service [11, 16, 20]. Service providers should also make follow-up actions available based on the answers to the questionnaires, and perhaps more time in follow-up supports should be spared to women who reported mental health problems. It may be difficult for women to answer PREMs questions honestly if they do not know who will see the answer, how the answer will be processed, or what will happen to them if they answer negatively [12, 16, 34, 35]. Thus, the process of how the information is used, and by whom, should be clearly explained to women before asking the questions. In addition, women should be given a chance to answer PREMs questions anonymously, as they may feel hesitant to give negative feedback directly to the professional with whom they interact. Similar arguments regarding anonymity can be found in two studies from the Netherlands [11, 12]. It is also suggested that the service quality of different providers or experiences of different service events should be assessed separately.

### Strength, limitations and future study

This is the first study in Finland or the Nordic area to explore pregnant and postpartum women’s attitudes and views toward the use of ICHOM-PCB-PRMs in public maternity care. We conducted in-depth interviews with the women to obtain deep insights. This differs from the approach of Laureij et al. (2019) [12], who asked women to simply score the measures for importance; and Depla et al. (2020) [11], who conducted a survey to explore women’s agreement with the use of the PCB set. By studying women’s perspectives, this study furthered our previous effort of understanding professionals’ perspectives towards the introduction of ICHOM-PCB-PRMs into Finnish maternity care pathway [20] and developed a broader view on the applicability and feasibility of using ICHOM-PCB-PRMs in Finland. We developed the interview guide, topics, and themes on the basis of our previous work of interviewing professionals. By applying similar interview topics, themes, data collection and analysis strategies, these two studies could be compared, so that we could observe the agreements and disagreements between professionals and women. In a care relationship, a caregiver is a professional with their own goals, rights, and responsibilities that may affect the communication and interaction between the medical professional and the client, while the client’s goals and expectations may be incompatible with these rules. Thus, to compare professional’s views with women’s views is important, especially in a patient-centered care.

Besides using texts and tables, we also used numbers and percentages in reporting the results for making the study transparent and providing a preliminary picture of women’s opinions on PRMs. But the results from this qualitative study cannot be completely transferred to a broader population. We conducted voluntary-based interviews, which might also affect the generalizability of the results, as it is possible that women who have a more positive attitude towards sharing their views are more likely to participate in the interviews. In addition, for each theme under the topic of women’s evaluation on ICHOM-PCB-PRMs, we only reported in the paper the comments mentioned by at least five (22.73%), considering the space of reporting and following the way we did in our previous study [20] where we also reported the points that was mentioned by at least five participants, 25% of all participants. We acknowledge that other comments mentioned by less than five participants could be also important but missed in this presented study. Furthermore, cultural and ethnic background was not considered in this study. We only recruited Finnish women from HUS area, and women living in Finland but with a non-Finnish background or from other health districts who might have different perspectives were missing from this study. This study provided a preliminary view on women’s perspectives regarding PRMs, and fail to deepen the understanding by systematically comparing opinions of women with different background (e.g. gravidity and parity) as in this study women express their thoughts not just based on their presented conditions but also based on their previous or imaged conditions. Thus, further research with improved study design is warranted. An expanded sample with women from other health districts and other cultural/ethnic backgrounds is also needed to improve the trustworthiness and transferability of our findings. With more comprehensive and solid evidence on the applicability and feasibility of ICHOM-PCB-PRMs in the Finnish context, we could develop a minimum set of PRMs based on ICHOM-PCB-PRMs for local use, set up a pilot to collect PRMs data from women with the developed set, and explore the experiences and impact of using PRMs in routine maternity care. Our research could help in developing guidelines for implementing PRMs in routine maternity care and add knowledge to the general knowledge base for implementing PRMs.

## Conclusion

This study explored the acceptability of ICHOM-PCB-PRMs in Finland and investigated the feasibility of systematically using PRMs in routine maternity care from women’s perspectives. It provided insights and experiences on the adoption and implementation of standard PRMs in routine maternity care. The study revealed that introducing ICHOM-PCB-PRMs is possible, but nationally interesting measures and questions could be added and local adaptation of the measures and questions is necessary. Measures for issues like preparation for birth, burden of childcare, caesarean experience and family situation should be added. Systematic implementation of PRMs may require some efforts. In this study, some benefits of using PRMs in local maternity facilities were identified. Possible solutions for implementing PRMs in local maternity care have been suggested. Careful consideration is needed regarding how and when PRMs questions are asked for enhancing more accurate and honest answers and minimizing women feeling judged, embarrassed, or offended. Follow-up care should be available according to women’s responses and needs.

### Electronic supplementary material

Below is the link to the electronic supplementary material.

Supplementary Material 1

Supplementary Material 2

Supplementary Material 3

## Data Availability

The dataset generated and analysed for this study is not publicly available due to the restrictions claimed in the research permissions and letter to interviewees. Data are however available from the authors upon reasonable request and with permissions of ethical committees of corresponding hospital districts and municipalities. For requesting the access to data and concerning issues related to the data, please contact with the corresponding author.

## List of Abbreviations

ICHOM: The International Consortium for Health Outcomes Measurement
PCB set: The ICHOM Set of Patient-Centred Outcome Measures for Pregnancy and Childbirth
PRM: Patient-reported measure
ICHOM-PCB-PRMs: Standard PRMs defined in ICHOM PCB set
PROM: Patient-reported outcome measure
PREM: Patient-reported experience measure
Neuvola: Municipality-organized community-based maternity and child health clinics in Finland
HUS: Helsinki and Uusimaa Hospital District
ePROMs: Electronic Patient Reported Outcome Measures
ePREMs: Electronic Patient Reported Experience Measures
