# Supplementary material for: Introducing standard patient-reported measures (PRMs) into routine maternity care: A pre-implementation qualitative study on women’s perspectives in Finland
Source: BMC Health Serv Res. 2023 Aug 10;23:845. doi: 10.1186/s12913-023-09818-5 (PMC10413725; doi:10.1186/s12913-023-09818-5)
Supplement: Supplementary file 1 — Supplementary Material 1 [file 12913_2023_9818_MOESM1_ESM.docx]

**Additional file 1. Patient-reported measures and questions defined in ICHOM PCB set**

*Source:*

*1. ICHOM PREGNANCY & CHILDBIRTH DATA COLLECTION REFERENCE GUIDE V1.0.3;*

*2. Nijagal MA, Wissig S, Stowell C, Olson E, Amer-Wahlin I, Bonsel G, et al. Standardized outcome measures for pregnancy and childbirth, an ICHOM proposal. BMC Health Serv Res. 2018;18(1):953-64.*

*3. Laureij LT, Been JV, Lugtenberg M, Ernst-Smelt HE, Franx A, Hazelzet JA, et al. Exploring the applicability of the pregnancy and childbirth outcome set: A mixed methods study. Patient Educ Couns. 2019;103(3):642-51.*

*Timeline:*


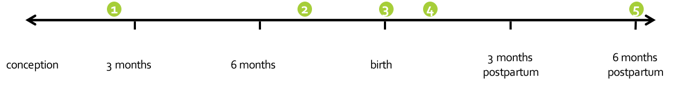


*Content:*

| **Patient reported information** | **Description** | **instruments/ items/questions** | **Time points of data collection** |
| --- | --- | --- | --- |
| Patient reported case-mix variables | Demographic factors and obstetric and medical history | 1. Education. Please indicate the highest level of schooling completed. [0 = None, 1= Primary, 2 = Secondary, 3 = Tertiary (university or equivalent)] 2. Race/ethnicity as defined locally. Varies by country and should be determined by country (not for cross country comparison). 3. Social Supports (SIMSS: Single Item Measure of Social Supports), How many people do you have near you that you can readily count on for help in time of difficulty such as to watch over children or pets, give rides to the hospital or store, or help when you are sick? [0 = 0, 1 = 1, 2 = 2 – 5, 3 = 6 - 10] 4. Parity. Have you given birth before? This includes both vaginal births and Cesarean sections (operations to remove your baby from your abdomen). Please do not count miscarriages or births that happened before 20weeks (5months) of pregnancy. [0 = No, 1 = Yes] 5. Obstetric history. If you have been pregnant before, have you experienced any of the following in previous pregnancies? Please mark all that apply. [0 = This is my first pregnancy, 1 = A baby born early, more than 3 weeks before his or her due date, 2 = Bleeding so much during pregnancy, birth, or after giving birth that you needed to be given blood, 3 = A cesarean section (operation to remove your baby through your abdomen), 4 = Loss of a pregnancy after 20 weeks (5 months) of pregnancy] 6. Medical history. BEFORE you got pregnant, did a doctor, nurse, or other health worker tell you that you had any of the following health conditions? Please mark all that apply. [0 = None, 1 = Diabetes, 2 = High blood pressure or hypertension, 3 = A mental health disorder such as depression, anxiety, bipolar disorder or schizophrenia] 7. Multiple gestations. Are you pregnant with: [1 = One baby, 2 = Two babies (twins), 3 = Three or more babies (triplets or higher)] 8. Body height. How tall are you? Indicate height in centimeters or inches. Also indicate units of height. Height and weight are used to calculate BMI. 9. Body weight. How much did you weigh IMMEDIATELY before your pregnancy? Indicate weight in kilograms or pounds. Also indicate units of weight. Height and weight are used to calculate BMI. | T1 |
| PROMs- health related quality of life | Perceived quality of life | Tracked via the [PROMIS Global10: Patient Reported Outcomes Measurement System Global10]   1. In the past few weeks... In general, would you say your health is:…  [5 = Excellent; 4 = Very good; 3 = Good; 2 = Fair; 1 = Poor] 2. In the past few weeks... In general, would you say your quality of life is:…   [5 = Excellent; 4 = Very good; 3 = Good; 2 = Fair; 1 = Poor] 3. In the past few weeks... In general, how would you rate your physical health?  [5 = Excellent; 4 = Very good; 3 = Good; 2 = Fair; 1 = Poor] 4. In the past few weeks... In general, how would you rate your mental health, including your mood and your ability to think? [5 = Excellent; 4 = Very good; 3 = Good; 2 = Fair; 1 = Poor] 5. In the past few weeks... In general, how would you rate your satisfaction with your social activities and relationships? [5 = Excellent; 4 = Very good; 3 = Good; 2 = Fair; 1 = Poor] 6. In the past few weeks... In general, please rate how well you carry out your usual social activities and roles.  (This includes activities at home, at work and in your community, and responsibilities as a parent, child, spouse, employee, friend, etc.)  [5 = Excellent; 4 = Very good; 3 = Good; 2 = Fair; 1 = Poor] 7. In the past few weeks… To what extent are you able to carry out your everyday physical activities such as walking, climbing stairs, carrying groceries, or moving a chair?  [5 = Excellent; 4 = Very good; 3 = Good; 2 = Fair; 1 = Poor] 8. In the past 7 days, how often have you been bothered by emotional problems such as feeling anxious, depressed or irritable?  [5 = Excellent; 4 = Very good; 3 = Good; 2 = Fair; 1 = Poor] 9. In the past 7 days... How would you rate your fatigue on average?  [5 = Excellent; 4 = Very good; 3 = Good; 2 = Fair; 1 = Poor] 10. How would you rate your pain on average?  [5 = Excellent; 4 = Very good; 3 = Good; 2 = Fair; 1 = Poor] | T1,T2,T4,T5 |
| PROMs- incontinence | Leaking of urine, stool, or gas during pregnancy or as a result of childbirth | Option to track via either the [ICIQ-SF: International Consultation on Incontinence Questionnaire-Short Form] or [Wexner]  [INCONTSCREEN]  In the past month, have you leaked urine, leaked stool or passed gas by accident? [1 = Yes, I leaked urine; 2 = Yes, I leaked stool or passed gas by accident; 3 = No, I did not leak urine, leak stool, or pass gas by accident]  [ICIQ--SF] *women scoring a 1 on INCONTSCREEN   1. How often do you leak urine? [0 = never; 1 = about once a week or less often; 2 = two or three times a week; 3 = about once a day; 4 = several time a day; 5 = all the time] 2. We would like to know how much urine you think leaks. How much urine do you usually leak (whether you wear protection or not)? [0 = none; 2 = a small amount; 4 = a moderate amount; 6 = a large amount] 3. Overall, how much does leaking urine interfere with your everyday life? [0 = not at all; 1 = 1; 2 = 2; 3 = 3; 4 = 4; 5 = 5; 6 = 6; 7 = 7; 8 = 8; 9 = 9; 10 = a great deal] 4. When does urine leak? (Please tick all that apply to you). [0 = Never - urine does not leak; 1 = Leaks before you can get to the toilet; 2 = Leaks when you cough or sneeze; 3 = Leaks when you are asleep; 4 = Leaks when you are physically active/exercising; 5 = Leaks when you have finished urinating and are dressed; 6 = Leaks for no obvious reason; 7 = Leaks all the time]   [Wexner] *women scoring a 2 on INCONTSCREEN   1. How often do you have accidents to solid, well-formed stool? [0 = Never; 1 = Less than once per month; 2 = Less than once/week & greater than once/month; 3 = Less than once/day & greater than once/month; 4 = Once a day or more than once a day] 2. How often do you have accidents to liquid stool/diarrhea? [0 = Never; 1 = Less than once per month; 2 = Less than once/week & greater than once/month; 3 = Less than once/day & greater than once/month; 4 = Once a day or more than once a day] 3. How often does the gas escape without your knowledge or control? [0 = Never; 1 = Less than once per month; 2 = Less than once/week & greater than once/month; 3 = Less than once/day & greater than once/month; 4 = Once a day or more than once a day] 4. How often do you wear a pad/depends or change underwear? [0 = Never; 1 = Less than once per month; 2 = Less than once/week & greater than once/month; 3 = Less than once/day & greater than once/month; 4 = Once a day or more than once a day] 5. How much do the above answers alter your lifestyle or activities? [0 = Never; 1 = Less than once per month; 2 = Less than once/week & greater than once/month; 3 = Less than once/day & greater than once/month; 4 = Once a day or more than once a day] | T1,T2,T4,T5 |
| PROMs- pain with intercourse | Having pain during sex as a result of childbirth | Tracked via [PROMIS SFFAC102: Patient Reported Outcomes Measurement Information System Sexual Function and Satisfaction]  In the past 30 days, how much has pain affected your satisfaction with your sex life? [0 = Have not had pain in the past 30 days; 1 = Not at all; 2 = A little bit; 3 = Somewhat; 4 = Quite a bit; 5 = Very much] | T1,T4,T5 |
| PROMs- confidence with role as a mother | Confidence of a woman regarding looking after her baby | [ROLECONF]  (when he or she is born) How confident do you feel about looking after your baby?  [1=Not at all confident; 2=Not very confident; 3=Somewhat confident; 4=Confident; 5=Very confident] | T1,T4,T5 |
| PROMs- mother-infant attachment | Having a bonding with your new baby, and feelings for the newborn in the first few weeks | Tracked via the [MIBS: Mother-Infant Bonding Scale]  [MIBS]  These questions are about your feelings for your child.  Please make a tick against each word in the box that best describes how you feel NOW*/felt in the FIRST FEW WEEKS after birth**.  (*delete if administering tool in postpartum period; **delete  if administering tool at birth)   1. Loving, 0 = Very much, 1 = A lot, 2 = A little, 3 = Not at all 2. Resentful, 0 = Very much, 1 = A lot, 2 = A little, 3 = Not at all 3. Neutral or felt nothing, 0 = Very much, 1 = A lot, 2 = A little, 3 = Not at all 4. Joyful, 0 = Very much, 1 = A lot, 2 = A little, 3 = Not at all 5. Dislike, 0 = Very much, 1 = A lot, 2 = A little, 3 = Not at all 6. Protective, 0 = Very much, 1 = A lot, 2 = A little, 3 = Not at all 7. Disappointed, 0 = Very much, 1 = A lot, 2 = A little, 3 = Not at all 8. Aggressive, 0 = Very much, 1 = A lot, 2 = A little, 3 = Not at all | T3,T4 |
| PROMs- maternal confidence with breastfeeding | Intention and confidence of a women regarding breastfeeding the baby | Option to track via the [BSES-SF: Breastfeeding Self-Efficacy Scale-Short Form]  [BFINTENT] (3rd trimester). Do you plan to breastfeed your baby when he or she is born? 0 = No, 1 = Yes  [BFCONFID] *women scoring a 1 on BFINTENT in 3rd trimester OR 1 or 2 on BFSUCCESS at birth, postpartum checkup, or 6 months postpartum.  How confident do you feel about breastfeeding? [1 = Not at all confident; 2 = Not very confident; 3 = Somewhat confident; 4 = Confident; 5 = Very confident]  [BSES-SF] *Optional for women scoring a 3 or lower on BFCONFID   1. I can always determine that my baby is getting enough milk. [1 = Not at all confident; 2 = Not very confident; 3 = Sometimes confident; 4 = Confident; 5 = Very confident] 2. I can always successfully cope with breastfeeding like I have with other challenging tasks. [1 = Not at all confident; 2 = Not very confident; 3 = Sometimes confident; 4 = Confident; 5 = Very confident] 3. I can always breastfeed my baby without using formula as a supplement. [1 = Not at all confident; 2 = Not very confident; 3 = Sometimes confident; 4 = Confident; 5 = Very confident] 4. I can always ensure that my baby is properly latched on for the whole feeding. [1 = Not at all confident; 2 = Not very confident; 3 = Sometimes confident; 4 = Confident; 5 = Very confident] 5. I can always manage the breastfeeding situation to my satisfaction. [1 = Not at all confident; 2 = Not very confident; 3 = Sometimes confident; 4 = Confident; 5 = Very confident] 6. I can always manage to breastfeed even if my baby is crying. [1 = Not at all confident; 2 = Not very confident; 3 = Sometimes confident; 4 = Confident; 5 = Very confident] 7. I can always keep wanting to breastfeed. [1 = Not at all confident; 2 = Not very confident; 3 = Sometimes confident; 4 = Confident; 5 = Very confident] 8. I can always comfortably breastfeed with my family members present. [1 = Not at all confident; 2 = Not very confident; 3 = Sometimes confident; 4 = Confident; 5 = Very confident] 9. I can always be satisfied with my breastfeeding experience. [1 = Not at all confident; 2 = Not very confident; 3 = Sometimes confident; 4 = Confident; 5 = Very confident] 10. I can always deal with the fact that breastfeeding can be time-consuming. [1 = Not at all confident; 2 = Not very confident; 3 = Sometimes confident; 4 = Confident; 5 = Very confident] 11. I can always finish feeding my baby on one breast before switching to the other breast. [1 = Not at all confident; 2 = Not very confident; 3 = Sometimes confident; 4 = Confident; 5 = Very confident] 12. I can always continue to breastfeed my baby for every feeding. [1 = Not at all confident; 2 = Not very confident; 3 = Sometimes confident; 4 = Confident; 5 = Very confident] 13. I can always manage to keep up with my baby's breastfeeding demands. [1 = Not at all confident; 2 = Not very confident; 3 = Sometimes confident; 4 = Confident; 5 = Very confident] 14. I can always tell when my baby is finished breastfeeding. [1 = Not at all confident; 2 = Not very confident; 3 = Sometimes confident; 4 = Confident; 5 = Very confident] | T2,T3,T4 |
| PROMs- success with breastfeeding | Duration of breastfeeding, continuation of breastfeeding, ideally until the baby is 6 months old | [BFSUCCESS] Please indicate how you are feeding your baby:  1=My baby has received only breast milk in the past 7 days.  2=My baby has received a combination of breast milk, formula, or water in the past 7 days.  3=My baby has received only formula, water, or other liquids but not breast milk in the past 7 days. | T3,T4,T5 |
| PROMs- postpartum depression | Clinically detecting depressive symptoms | Assessed via the [PHQ-2: Patient Health Questionnaire-2] with optional follow-up with the [EPDS: Edinburgh Postnatal Depression Scale]  [PHQ-2] Screening with PHQ1 and PHQ2 in PHQ-9  Over the past 2 weeks, how often have you been bothered by any of the following problems?   1. Little interest or pleasure in doing things [1 = Not at all; 2 = Several days; 3 = More than half the days; 4 = Nearly every day] 2. Feeling down, depressed or hopeless [1 = Not at all; 2 = Several days; 3 = More than half the days; 4 = Nearly every day]   [EPDS]  As you are pregnant or have recently had a baby, we would like to know how you are feeling. Please check the answer that comes closest to how you have felt IN THE PAST 7 DAYS, not just how you feel today.   1. *Optional for women scoring a 3 or higher on the PHQ-2. I have been able to see the funny side of things. [0 = As much as I always could; 1 = Not quite so much now; 2 = Definitely not so much now; 3 = Not at all] 2. *Optional for women scoring a 3 or higher on the PHQ-2. I have looked forward with enjoyment to things. [0 = As much as I ever did; 1 = Rather less than I used to; 2 = Definitely less than I used to; 3 = Hardly at all] 3. *Optional for women scoring a 3 or higher on the PHQ-2. I have blamed myself unnecessarily when things when wrong. [0 = Yes, most of the time; 1 = Yes, some of the time; 2 = Not very often; 3 = No, never] 4. *Optional for women scoring a 3 or higher on the PHQ-2. I have been anxious or worried for no good reason. [0 = No, not at all; 1 = Hardly ever; 2 = Yes, sometimes; 3 = Yes, very often] 5. *Optional for women scoring a 3 or higher on the PHQ-2. I have felt scared or panicky for no very good reason. [0 = Yes, quite a lot; 1 = Yes, sometimes; 2 = No, not much; 3 = No, not at all] 6. *Optional for women scoring a 3 or higher on the PHQ-2. Things have been getting on top of me. [0 = Yes, most of the time; 1 = Yes, quite often; 2 = Not very often; 3 = No, not at all] 7. *Optional for women scoring a 3 or higher on the PHQ-2. I have been so unhappy that I have difficulty sleeping. [0 = Yes, most of the time; 1 = Yes, quite often; 2 = Not very often; 3 = No, not at all] 8. *Optional for women scoring a 3 or higher on the PHQ-2. I have felt sad or miserable. [0 = Yes, most of the time; 1 = Yes, quite often; 2 = Not very often; 3 = No, not at all] 9. *Optional for women scoring a 3 or higher on the PHQ-2. I have been so unhappy that I have been crying. [0 = Yes, most of the time; 1 = Yes, quite often; 2 = Only occasionally; 3 = No, never] 10. *Optional for women scoring a 3 or higher on the PHQ-2. The thought of harming myself has occurred to me. [0 = Yes, quite often; 1 = Sometimes; 2 = Hardly ever; 3 = Never] | [PHQ-2] T1,T2,T4,T5  [EPDS]  T4,T5 |
| PREMs- satisfaction with the result of care | Degree of satisfaction of a woman with results of received care | [CARESAT]  How satisfied are you with the results of your care during your pregnancy?* "during your labor and the birth of your baby" or "in the months after your baby was born" 0 = Very unsatisfied, 1 = Unsatisfied , 2 = Neither satisfied nor dissatisfied, 3 = Satisfied, 4 = Very satisfied | T2,T4,T5 |
| PREMs- confidence as an active participant in healthcare decisions | Confidence as an active participant in decisions; To measure whether health care providers 1) gave information about women’s choices for maternity care, 2) gave enough information at the right times to help women made decisions, 3) inspired women’s confidence and trust | [HCR]Thinking about your care “during your pregnancy”/"during your labor and the birth of your baby"/"in the months after your baby was born"   1. Were you given information about your choices for maternity care? 0 = No, 1= To some extent, 2 = Yes 2. Were you given enough information to help you decide about your care? 0 = No, 1= To some extent, 2 = Yes 3. Were you given information at the right time to help you decide about your care? 0 = No, 1 = To some extent, 2 = Yes | T2,T4,T5 |
| PREMs- confidence in healthcare providers | Perceived confidence in healthcare professionals | [HCR]Thinking about your care “during your pregnancy”/"during your labor and the birth of your baby"/"in the months after your baby was born"  1. Did you have confidence and trust in the staff caring for you? 0 = No, 1 = To some extent, 2 = Yes | T2,T4,T5 |
| PREMs- birth experience | Assessment of a woman’s experiences during labour and birth | Tracked via [BSS_R: Birth Satisfaction Scale-Revised]  [BSS_R]  Please respond to the following statements: 4 = Strongly Agree, 3 = Agree, 2 = Neither Agree or Disagree, 1 = Disagree, 0 = Strongly Disagree   1. I came through childbirth virtually unscathed. 2. I thought my labour was excessively long. 3. The delivery room staff encouraged me to take decisions about how I wanted my birth to progress. 4. I felt very anxious during my labour and birth. 5. I felt well supported by staff during my labour and birth. 6. The staff communicated well with me during labour. 7. I found giving birth a distressing experience. 8. I felt out of control during my birth experience. 9. I was not distressed at all during labour. 10. The delivery room was clean and hygienic. | T4 |
