# Supplementary material for: Introducing standard patient-reported measures (PRMs) into routine maternity care: A pre-implementation qualitative study on women’s perspectives in Finland
Source: BMC Health Serv Res. 2023 Aug 10;23:845. doi: 10.1186/s12913-023-09818-5 (PMC10413725; doi:10.1186/s12913-023-09818-5)
Supplement: Supplementary file 2 — Supplementary Material 2 [file 12913_2023_9818_MOESM2_ESM.docx]

**Additional file 2. A structure of data collection and analysis with predefined themes**

| **Topics** | **Themes** |
| --- | --- |
| women’s evaluation of ICHOM-PCB-PRMs^*^ | importance and relevance |
|  | time points of data collection |
|  | quality of questions |
|  | willingness to answer |
| women’s views on implementing PRMs^+^ in routine maternity care | expected benefits of implementing PRMs or motivations to respond to PRMs questions |
|  | possible difficulties or risks |
|  | preferred practices or conditions |

^*^ ICHOM-PCB-PRMs: standard patient-reported measures defined in Patient-Centered Outcome Measures for Pregnancy and Childbirth set developed by The International Consortium for Health Outcomes Measurement

^+^PRMs: patient-reported measures
