# Supplementary material for: Introducing standard patient-reported measures (PRMs) into routine maternity care: A pre-implementation qualitative study on women’s perspectives in Finland
Source: BMC Health Serv Res. 2023 Aug 10;23:845. doi: 10.1186/s12913-023-09818-5 (PMC10413725; doi:10.1186/s12913-023-09818-5)
Supplement: Supplementary file 3 — Supplementary Material 3 [file 12913_2023_9818_MOESM3_ESM.docx]

**Additional file 3. Interview protocol and sample questions**

## **Part 1- Pregnancy experiences and expectations (20 mins)**

- How do you feel now?
- Could you please describe you experiences (e.g. memorable events, feelings, perceptions, expectations, thoughts, worries. etc.), starting from when you found you were pregnant?
- Based on your experiences now, what could be a good life during pregnancy? what could be good birth experiences? what kind of family life is good with a newborn?
- What kinds of challenges are/were you facing and how you cope with those challenges?
- What kind of choices and decisions have you made during pregnancy and childbirth? Could you please describe the decision-making experiences?
- What medical treatments and/or health services that you have received after you found you were pregnancy? How do you feel? What thoughts and comments you have regarding the care?

## **Part 2- Evaluation on the following self-reported measures (30-40 mins)**

Please assess, from your own view, whether the following self-reported measures are understandable, important, appropriate, and easy to answer.

Here self-reported measures refer to questionnaires where people are asked to report directly on their health status, e.g. physical conditions and psychological feelings, and also express their perceptions and views of the received care.

We go through the following self-reported measures together one by one.

(Researcher selects measures that need to be evaluated during interview according to the woman’s pregnancy weeks or postpartum stage.)

- For women during pregnancy week 26-27, measure 1, 2, 3, 4, 5, 6, 7, 9, 10, 11, 12
- For women during pregnancy week 35-36, measure 2, 3, 4, 5, 6, 7, 9, 10, 11, 12
- For women during the 3rd -4th month postpartum, measure 2, 3, 4, 5, 6, 7, 8, 9, 10, 11, 12, 13
- For women during the 6th – 7th month postpartum 2, 3, 4, 5, 6, 7, 8, 9, 10, 11, 12
- For women during the 12th -13th month postpartum 2, 3, 4, 5, 6, 7, 8, 9, 10, 11, 12

**Sample interview questions for each self-reported measure:**

- Do you think if these are important and meaningful questions to be asked during pregnancy and childbirth? Why important? / why not important?
- Do you think if these questions should be asked at this stage as you are at pregnancy week X or postpartum week X? Why it is not good time/ why it is good time? Do you think when these questions should be asked?
- Do you think if these questions are understandable? / Do you have any difficulty in understanding on these questions? Which one or ones?
- Do you think if it is difficult to answer these questions? / Do you have any difficulty in answering these questions? Which one or ones?
- Do you think the response options fit the questions? Which one or ones not appropriate?
- Did you wonder about anything when reading these questions? What you were wondering?
- Are you willing to give answers to these questions? why if not?
- What other comments you have?

### **Measure 1: Self-reported background Information**

1. Education level. Please indicated the highest level of schooling completed. [0 = None, 1 = Primary, 2 = Secondary, 3 = Tertiary (university or equivalent)]
2. Social Support. How many people do you have near you that you can readily count on for help in time of difficulty such as to watch over children or pets, give rides to the hospital or store, or help when you are sick? [0 = 0, 1 = 1, 2 = 2 – 5, 3 = 6 - 10]
3. Parity. Have you given birth before? This includes both vaginal births and Cesarean sections (operations to remove the baby from abdomen). Please do not count miscarriages or births that happened before 20 weeks (5 months) of pregnancy. [0 = No, 1 = Yes]
4. Medical history. BEFORE you got pregnant, did a doctor, nurse, or other health worker tell you that you had any of the following health conditions? Please mark all that apply. [0 = None, 1 = Diabetes, 2 = High blood pressure or hypertension, 3 = A mental health disorder such as depression, anxiety, bipolar disorder or schizophrenia]
5. Body height. How tall are you?
6. Body weight. How much did you weigh IMMEDIATELY before your pregnancy?

**Interview questions for this measure:**

- Do you think if these are important and meaningful questions to be asked during pregnancy and childbirth? Why important? / why not important?
- Do you think if these questions should be asked at this stage as you are at pregnancy week X or postpartum week X? Why it is not good time/ why it is good time? Do you think when these questions should be asked?
- Do you think if these questions are understandable? / Do you have any difficulty in understanding on these questions? Which one or ones?
- Do you think if it is difficult to answer these questions? / Do you have any difficulty in answering these questions? Which one or ones?
- Do you think the response options fit the questions? Which one or ones not appropriate?
- Did you wonder about anything when reading these questions? What you were wondering?
- Are you willing to give answers to these questions? why if not?
- What other comments you have?

### **Measure 2: health related quality of life**

[PROMIS Global10]

1. In the past few weeks... In general, would you say your health is:… [5 = Excellent; 4 = Very good; 3 = Good; 2 = Fair; 1 = Poor]
2. In the past few weeks... In general, would you say your quality of life is:… [5 = Excellent; 4 = Very good; 3 = Good; 2 = Fair; 1 = Poor]
3. In the past few weeks... In general, how would you rate your physical health? [5 = Excellent; 4 = Very good; 3 = Good; 2 = Fair; 1 = Poor]
4. In the past few weeks... In general, how would you rate your mental health, including your mood and your ability to think? [5 = Excellent; 4 = Very good; 3 = Good; 2 = Fair; 1 = Poor]
5. In the past few weeks... In general, how would you rate your satisfaction with your social activities and relationships? [5 = Excellent; 4 = Very good; 3 = Good; 2 = Fair; 1 = Poor]
6. In the past few weeks... In general, please rate how well you carry out your usual social activities and roles. (This includes activities at home, at work and in your community, and responsibilities as a parent, child, spouse, employee, friend, etc.) [5 = Excellent; 4 = Very good; 3 = Good; 2 = Fair; 1 = Poor]
7. In the past few weeks… To what extent are you able to carry out your everyday physical activities such as walking, climbing stairs, carrying groceries, or moving a chair? [5 = Excellent; 4 = Very good; 3 = Good; 2 = Fair; 1 = Poor]
8. In the past 7 days, how often have you been bothered by emotional problems such as feeling anxious, depressed or irritable? [5 = Excellent; 4 = Very good; 3 = Good; 2 = Fair; 1 = Poor]
9. In the past 7 days... How would you rate your fatigue on average? [5 = Excellent; 4 = Very good; 3 = Good; 2 = Fair; 1 = Poor]
10. How would you rate your pain on average? [5 = Excellent; 4 = Very good; 3 = Good; 2 = Fair; 1 = Poor]

**Interview questions for this measure:**

- Do you think if these are important and meaningful questions to be asked during pregnancy and childbirth? Why important? / why not important?
- Do you think if these questions should be asked at this stage as you are at pregnancy week X or postpartum week X? Why it is not good time/ why it is good time? Do you think when these questions should be asked?
- Do you think if these questions are understandable? / Do you have any difficulty in understanding on these questions? Which one or ones?
- Do you think if it is difficult to answer these questions? / Do you have any difficulty in answering these questions? Which one or ones?
- Do you think the response options fit the questions? Which one or ones not appropriate?
- Did you wonder about anything when reading these questions? What you were wondering?
- Are you willing to give answers to these questions? why if not?
- What other comments you have?

### **Measure 3: incontinence**

In the past month, have you leaked urine, leaked stool or passed gas by accident? [1 = Yes, I leaked urine; 2 = Yes, I leaked stool or passed gas by accident; 3 = No, I did not leak urine, leak stool, or pass gas by accident]

[ICIQ-SF: International Consultation on Incontinence Questionnaire-Short Form]

*women scoring a 1 on INCONTSCREEN

1. How often do you leak urine? [0 = never; 1 = about once a week or less often; 2 = two or three times a week; 3 = about once a day; 4 = several time a day; 5 = all the time]
2. We would like to know how much urine you think leaks. How much urine do you usually leak (whether you wear protection or not)? [0 = none; 2 = a small amount; 4 = a moderate amount; 6 = a large amount]
3. Overall, how much does leaking urine interfere with your everyday life? [0 = not at all; 1 = 1; 2 = 2; 3 = 3; 4 = 4; 5 = 5; 6 = 6; 7 = 7; 8 = 8; 9 = 9; 10 = a great deal]
4. When does urine leak? (Please tick all that apply to you). [0 = Never - urine does not leak; 1 = Leaks before you can get to the toilet; 2 = Leaks when you cough or sneeze; 3 = Leaks when you are asleep; 4 = Leaks when you are physically active/exercising; 5 = Leaks when you have finished urinating and are dressed; 6 = Leaks for no obvious reason; 7 = Leaks all the time]

[Wexner] *women scoring a 2 on INCONTSCREEN

1. How often do you have accidents to solid, well-formed stool? [0 = Never; 1 = Less than once per month; 2 = Less than once/week & greater than once/month; 3 = Less than once/day & greater than once/month; 4 = Once a day or more than once a day]
2. How often do you have accidents to liquid stool/diarrhea? [0 = Never; 1 = Less than once per month; 2 = Less than once/week & greater than once/month; 3 = Less than once/day & greater than once/month; 4 = Once a day or more than once a day]
3. How often does the gas escape without your knowledge or control? [0 = Never; 1 = Less than once per month; 2 = Less than once/week & greater than once/month; 3 = Less than once/day & greater than once/month; 4 = Once a day or more than once a day]
4. How often do you wear a pad/depends or change underwear? [0 = Never; 1 = Less than once per month; 2 = Less than once/week & greater than once/month; 3 = Less than once/day & greater than once/month; 4 = Once a day or more than once a day]
5. How much do the above answers alter your lifestyle or activities? [0 = Never; 1 = Less than once per month; 2 = Less than once/week & greater than once/month; 3 = Less than once/day & greater than once/month; 4 = Once a day or more than once a day]

**Interview questions for this measure:**

- Do you think if these are important and meaningful questions to be asked during pregnancy and childbirth? Why important? / why not important?
- Do you think if these questions should be asked at this stage as you are at pregnancy week X or postpartum week X? Why it is not good time/ why it is good time? Do you think when these questions should be asked?
- Do you think if these questions are understandable? / Do you have any difficulty in understanding on these questions? Which one or ones?
- Do you think if it is difficult to answer these questions? / Do you have any difficulty in answering these questions? Which one or ones?
- Do you think the response options fit the questions? Which one or ones not appropriate?
- Did you wonder about anything when reading these questions? What you were wondering?
- Are you willing to give answers to these questions? why if not?
- What other comments you have?

### **Measure 4: pain with intercourse**

[PROMIS SFFAC102] In the past 30 days, how much has pain affected your satisfaction with your sex life? [0 = Have not had pain in the past 30 days; 1 = Not at all; 2 = A little bit; 3 = Somewhat; 4 = Quite a bit; 5 = Very much]

**Interview questions for this measure:**

- Do you think if these are important and meaningful questions to be asked during pregnancy and childbirth? Why important? / why not important?
- Do you think if these questions should be asked at this stage as you are at pregnancy week X or postpartum week X? Why it is not good time/ why it is good time? Do you think when these questions should be asked?
- Do you think if these questions are understandable? / Do you have any difficulty in understanding on these questions? Which one or ones?
- Do you think if it is difficult to answer these questions? / Do you have any difficulty in answering these questions? Which one or ones?
- Do you think the response options fit the questions? Which one or ones not appropriate?
- Did you wonder about anything when reading these questions? What you were wondering?
- Are you willing to give answers to these questions? why if not?
- What other comments you have?

### **Measure 5: Confidence with role as a mother**

(when he or she is born) How confident do you feel about looking after your baby?

[1=Not at all confident; 2=Not very confident; 3=Somewhat confident; 4=Confident; 5=Very confident]

**Interview questions for this measure:**

- Do you think if these are important and meaningful questions to be asked during pregnancy and childbirth? Why important? / why not important?
- Do you think if these questions should be asked at this stage as you are at pregnancy week X or postpartum week X? Why it is not good time/ why it is good time? Do you think when these questions should be asked?
- Do you think if these questions are understandable? / Do you have any difficulty in understanding on these questions? Which one or ones?
- Do you think if it is difficult to answer these questions? / Do you have any difficulty in answering these questions? Which one or ones?
- Do you think the response options fit the questions? Which one or ones not appropriate?
- Did you wonder about anything when reading these questions? What you were wondering?
- Are you willing to give answers to these questions? why if not?
- What other comments you have?

### **Measure 6: mother-infant attachment**

[MIBS: Mother-Infant Bonding Scale] These questions are about your feelings for your child.

Please make a tick against each word in the box that best describes how you feel NOW/felt in the FIRST FEW WEEKS after birth

1. Loving, [0 = Very much, 1 = A lot, 2 = A little, 3 = Not at all]
2. Resentful, [0 = Very much, 1 = A lot, 2 = A little, 3 = Not at all]
3. Neutral or felt nothing, [0 = Very much, 1 = A lot, 2 = A little, 3 = Not at all]
4. Joyful, [0 = Very much, 1 = A lot, 2 = A little, 3 = Not at all]
5. Dislike, [0 = Very much, 1 = A lot, 2 = A little, 3 = Not at all]
6. Protective, [0 = Very much, 1 = A lot, 2 = A little, 3 = Not at all]
7. Disappointed, [0 = Very much, 1 = A lot, 2 = A little, 3 = Not at all]
8. Aggressive, [0 = Very much, 1 = A lot, 2 = A little, 3 = Not at all]

**Interview questions for this measure:**

- Do you think if these are important and meaningful questions to be asked during pregnancy and childbirth? Why important? / why not important?
- Do you think if these questions should be asked at this stage as you are at pregnancy week X or postpartum week X? Why it is not good time/ why it is good time? Do you think when these questions should be asked?
- Do you think if these questions are understandable? / Do you have any difficulty in understanding on these questions? Which one or ones?
- Do you think if it is difficult to answer these questions? / Do you have any difficulty in answering these questions? Which one or ones?
- Do you think the response options fit the questions? Which one or ones not appropriate?
- Did you wonder about anything when reading these questions? What you were wondering?
- Are you willing to give answers to these questions? why if not?
- What other comments you have?

### **Measure 7: maternal confidence with breastfeeding**

[BFINTENT] Do you plan to breastfeed your baby when he or she is born? 0 = No, 1 = Yes

[BFCONFID] *women scoring a 1 on BFINTENT in 3rd trimester OR 1 or 2 on BFSUCCESS at birth, postpartum checkup, or 6 months postpartum.

How confident do you feel about breastfeeding? [1 = Not at all confident; 2 = Not very confident; 3 = Somewhat confident; 4 = Confident; 5 = Very confident]

[BSES-SF: Breastfeeding Self-Efficacy Scale-Short Form] *Optional for women scoring a 3 or lower on BFCONFID

1. I can always determine that my baby is getting enough milk. [1 = Not at all confident; 2 = Not very confident; 3 = Sometimes confident; 4 = Confident; 5 = Very confident]
2. I can always successfully cope with breastfeeding like I have with other challenging tasks. [1 = Not at all confident; 2 = Not very confident; 3 = Sometimes confident; 4 = Confident; 5 = Very confident]
3. I can always breastfeed my baby without using formula as a supplement. [1 = Not at all confident; 2 = Not very confident; 3 = Sometimes confident; 4 = Confident; 5 = Very confident]
4. I can always ensure that my baby is properly latched on for the whole feeding. [1 = Not at all confident; 2 = Not very confident; 3 = Sometimes confident; 4 = Confident; 5 = Very confident]
5. I can always manage the breastfeeding situation to my satisfaction. [1 = Not at all confident; 2 = Not very confident; 3 = Sometimes confident; 4 = Confident; 5 = Very confident]
6. I can always manage to breastfeed even if my baby is crying. [1 = Not at all confident; 2 = Not very confident; 3 = Sometimes confident; 4 = Confident; 5 = Very confident]
7. I can always keep wanting to breastfeed. [1 = Not at all confident; 2 = Not very confident; 3 = Sometimes confident; 4 = Confident; 5 = Very confident]
8. I can always comfortably breastfeed with my family members present. [1 = Not at all confident; 2 = Not very confident; 3 = Sometimes confident; 4 = Confident; 5 = Very confident]
9. I can always be satisfied with my breastfeeding experience. [1 = Not at all confident; 2 = Not very confident; 3 = Sometimes confident; 4 = Confident; 5 = Very confident]
10. I can always deal with the fact that breastfeeding can be time-consuming. [1 = Not at all confident; 2 = Not very confident; 3 = Sometimes confident; 4 = Confident; 5 = Very confident]
11. I can always finish feeding my baby on one breast before switching to the other breast. [1 = Not at all confident; 2 = Not very confident; 3 = Sometimes confident; 4 = Confident; 5 = Very confident]
12. I can always continue to breastfeed my baby for every feeding. [1 = Not at all confident; 2 = Not very confident; 3 = Sometimes confident; 4 = Confident; 5 = Very confident]
13. I can always manage to keep up with my baby's breastfeeding demands. [1 = Not at all confident; 2 = Not very confident; 3 = Sometimes confident; 4 = Confident; 5 = Very confident]
14. I can always tell when my baby is finished breastfeeding. [1 = Not at all confident; 2 = Not very confident; 3 = Sometimes confident; 4 = Confident; 5 = Very confident]

**Interview questions for this measure:**

- Do you think if these are important and meaningful questions to be asked during pregnancy and childbirth? Why important? / why not important?
- Do you think if these questions should be asked at this stage as you are at pregnancy week X or postpartum week X? Why it is not good time/ why it is good time? Do you think when these questions should be asked?
- Do you think if these questions are understandable? / Do you have any difficulty in understanding on these questions? Which one or ones?
- Do you think if it is difficult to answer these questions? / Do you have any difficulty in answering these questions? Which one or ones?
- Do you think the response options fit the questions? Which one or ones not appropriate?
- Did you wonder about anything when reading these questions? What you were wondering?
- Are you willing to give answers to these questions? why if not?
- What other comments you have?

### **Measure 8: success with breastfeeding**

[BFSUCCESS] Please indicate how you are feeding your baby:

1. 1=My baby has received only breast milk in the past 7 days.
2. 2=My baby has received a combination of breast milk and formula in the past 7 days.
3. 3=My baby has received only formula or other liquids but not breast milk in the past 7 days.

**Interview questions for this measure:**

- Do you think if these are important and meaningful questions to be asked during pregnancy and childbirth? Why important? / why not important?
- Do you think if these questions should be asked at this stage as you are at pregnancy week X or postpartum week X? Why it is not good time/ why it is good time? Do you think when these questions should be asked?
- Do you think if these questions are understandable? / Do you have any difficulty in understanding on these questions? Which one or ones?
- Do you think if it is difficult to answer these questions? / Do you have any difficulty in answering these questions? Which one or ones?
- Do you think the response options fit the questions? Which one or ones not appropriate?
- Did you wonder about anything when reading these questions? What you were wondering?
- Are you willing to give answers to these questions? why if not?
- What other comments you have?

### **Measure 9: postpartum depression**

[PHQ-2: Patient Health Questionnaire-2] Over the past 2 weeks, how often have you been bothered by any of the following problems?

1. Little interest or pleasure in doing things [1 = Not at all; 2 = Several days; 3 = More than half the days; 4 = Nearly every day]
2. Feeling down, depressed or hopeless [1 = Not at all; 2 = Several days; 3 = More than half the days; 4 = Nearly every day]

[EPDS: Edinburgh Postnatal Depression Scale] As you are pregnant or have recently had a baby, we would like to know how you are feeling. Please check the answer that comes closest to how you have felt IN THE PAST 7 DAYS, not just how you feel today.

1. *Optional for women scoring a 3 or higher on the PHQ-2. I have been able to see the funny side of things. [0 = As much as I always could; 1 = Not quite so much now; 2 = Definitely not so much now; 3 = Not at all]
2. *Optional for women scoring a 3 or higher on the PHQ-2. I have looked forward with enjoyment to things. [0 = As much as I ever did; 1 = Rather less than I used to; 2 = Definitely less than I used to; 3 = Hardly at all]
3. *Optional for women scoring a 3 or higher on the PHQ-2. I have blamed myself unnecessarily when things when wrong. [0 = Yes, most of the time; 1 = Yes, some of the time; 2 = Not very often; 3 = No, never]
4. *Optional for women scoring a 3 or higher on the PHQ-2. I have been anxious or worried for no good reason. [0 = No, not at all; 1 = Hardly ever; 2 = Yes, sometimes; 3 = Yes, very often]
5. *Optional for women scoring a 3 or higher on the PHQ-2. I have felt scared or panicky for no very good reason. [0 = Yes, quite a lot; 1 = Yes, sometimes; 2 = No, not much; 3 = No, not at all]
6. *Optional for women scoring a 3 or higher on the PHQ-2. Things have been getting on top of me. [0 = Yes, most of the time; 1 = Yes, quite often; 2 = Not very often; 3 = No, not at all]
7. *Optional for women scoring a 3 or higher on the PHQ-2. I have been so unhappy that I have difficulty sleeping. [0 = Yes, most of the time; 1 = Yes, quite often; 2 = Not very often; 3 = No, not at all]
8. *Optional for women scoring a 3 or higher on the PHQ-2. I have felt sad or miserable. [0 = Yes, most of the time; 1 = Yes, quite often; 2 = Not very often; 3 = No, not at all]
9. *Optional for women scoring a 3 or higher on the PHQ-2. I have been so unhappy that I have been crying. [0 = Yes, most of the time; 1 = Yes, quite often; 2 = Only occasionally; 3 = No, never]
10. *Optional for women scoring a 3 or higher on the PHQ-2. The thought of harming myself has occurred to me. [0 = Yes, quite often; 1 = Sometimes; 2 = Hardly ever; 3 = Never]

**Interview questions for this measure:**

- Do you think if these are important and meaningful questions to be asked during pregnancy and childbirth? Why important? / why not important?
- Do you think if these questions should be asked at this stage as you are at pregnancy week X or postpartum week X? Why it is not good time/ why it is good time? Do you think when these questions should be asked?
- Do you think if these questions are understandable? / Do you have any difficulty in understanding on these questions? Which one or ones?
- Do you think if it is difficult to answer these questions? / Do you have any difficulty in answering these questions? Which one or ones?
- Do you think the response options fit the questions? Which one or ones not appropriate?
- Did you wonder about anything when reading these questions? What you were wondering?
- Are you willing to give answers to these questions? why if not?
- What other comments you have?

### **Measure 10: satisfaction with the result of care**

How satisfied are you with the results of your care “during your pregnancy?”, “during your labor and the birth of your baby” or “in the months after your baby was born” [0 = Very unsatisfied, 1 = Unsatisfied , 2 = Neither satisfied nor dissatisfied, 3 = Satisfied, 4 = Very satisfied]

**Interview questions for this measure:**

- Do you think if these are important and meaningful questions to be asked during pregnancy and childbirth? Why important? / why not important?
- Do you think if these questions should be asked at this stage as you are at pregnancy week X or postpartum week X? Why it is not good time/ why it is good time? Do you think when these questions should be asked?
- Do you think if these questions are understandable? / Do you have any difficulty in understanding on these questions? Which one or ones?
- Do you think if it is difficult to answer these questions? / Do you have any difficulty in answering these questions? Which one or ones?
- Do you think the response options fit the questions? Which one or ones not appropriate?
- Did you wonder about anything when reading these questions? What you were wondering?
- Are you willing to give answers to these questions? why if not?
- What other comments you have?

### **Measure 11: confidence as an active participant in healthcare decisions**

Thinking about your care “during your pregnancy”/"during your labor and the birth of your baby"/"in the months after your baby was born"

1. Were you given information about your choices for maternity care? [0 = No, 1= To some extent, 2 = Yes]
2. Were you given enough information to help you decide about your care? [0 = No, 1= To some extent, 2 = Yes]
3. Were you given information at the right time to help you decide about your care? [0 = No, 1 = To some extent, 2 = Yes]

**Interview questions for this measure:**

- Do you think if these are important and meaningful questions to be asked during pregnancy and childbirth? Why important? / why not important?
- Do you think if these questions should be asked at this stage as you are at pregnancy week X or postpartum week X? Why it is not good time/ why it is good time? Do you think when these questions should be asked?
- Do you think if these questions are understandable? / Do you have any difficulty in understanding on these questions? Which one or ones?
- Do you think if it is difficult to answer these questions? / Do you have any difficulty in answering these questions? Which one or ones?
- Do you think the response options fit the questions? Which one or ones not appropriate?
- Did you wonder about anything when reading these questions? What you were wondering?
- Are you willing to give answers to these questions? why if not?
- What other comments you have?

### **Measure 12: confidence in healthcare providers**

Thinking about your care “during your pregnancy”/"during your labor and the birth of your baby"/"in the months after your baby was born" Did you have confidence and trust in the staff caring for you? [0 = No, 1 = To some extent, 2 = Yes]

**Interview questions for this measure:**

- Do you think if these are important and meaningful questions to be asked during pregnancy and childbirth? Why important? / why not important?
- Do you think if these questions should be asked at this stage as you are at pregnancy week X or postpartum week X? Why it is not good time/ why it is good time? Do you think when these questions should be asked?
- Do you think if these questions are understandable? / Do you have any difficulty in understanding on these questions? Which one or ones?
- Do you think if it is difficult to answer these questions? / Do you have any difficulty in answering these questions? Which one or ones?
- Do you think the response options fit the questions? Which one or ones not appropriate?
- Did you wonder about anything when reading these questions? What you were wondering?
- Are you willing to give answers to these questions? why if not?
- What other comments you have?

### **Measure 13: birth experience**

[BSS_R: Birth Satisfaction Scale-Revised]

Please respond to the following statements: [4 = Strongly Agree, 3 = Agree, 2 = Neither Agree or Disagree, 1 = Disagree, 0 = Strongly Disagree]

1. I came through childbirth virtually unscathed. [4 = Strongly Agree, 3 = Agree, 2 = Neither Agree or Disagree, 1 = Disagree, 0 = Strongly Disagree]
2. I thought my labour was excessively long. [4 = Strongly Agree, 3 = Agree, 2 = Neither Agree or Disagree, 1 = Disagree, 0 = Strongly Disagree]
3. The delivery room staff encouraged me to take decisions about how I wanted my birth to progress. [4 = Strongly Agree, 3 = Agree, 2 = Neither Agree or Disagree, 1 = Disagree, 0 = Strongly Disagree]
4. I felt very anxious during my labour and birth. [4 = Strongly Agree, 3 = Agree, 2 = Neither Agree or Disagree, 1 = Disagree, 0 = Strongly Disagree]
5. I felt well supported by staff during my labour and birth. [4 = Strongly Agree, 3 = Agree, 2 = Neither Agree or Disagree, 1 = Disagree, 0 = Strongly Disagree]
6. The staff communicated well with me during labour. [4 = Strongly Agree, 3 = Agree, 2 = Neither Agree or Disagree, 1 = Disagree, 0 = Strongly Disagree]
7. I found giving birth a distressing experience. [4 = Strongly Agree, 3 = Agree, 2 = Neither Agree or Disagree, 1 = Disagree, 0 = Strongly Disagree]
8. I felt out of control during my birth experience. [4 = Strongly Agree, 3 = Agree, 2 = Neither Agree or Disagree, 1 = Disagree, 0 = Strongly Disagree]
9. I was not distressed at all during labour. [4 = Strongly Agree, 3 = Agree, 2 = Neither Agree or Disagree, 1 = Disagree, 0 = Strongly Disagree]
10. The delivery room was clean and hygienic. [4 = Strongly Agree, 3 = Agree, 2 = Neither Agree or Disagree, 1 = Disagree, 0 = Strongly Disagree]

**Interview questions for this measure:**

- Do you think if these are important and meaningful questions to be asked during pregnancy and childbirth? Why important? / why not important?
- Do you think if these questions should be asked at this stage as you are at pregnancy week X or postpartum week X? Why it is not good time/ why it is good time? Do you think when these questions should be asked?
- Do you think if these questions are understandable? / Do you have any difficulty in understanding on these questions? Which one or ones?
- Do you think if it is difficult to answer these questions? / Do you have any difficulty in answering these questions? Which one or ones?
- Do you think the response options fit the questions? Which one or ones not appropriate?
- Did you wonder about anything when reading these questions? What were you wondering?
- Are you willing to give answers to these questions? why if not?
- What other comments you have?

### **Other questions**

- What are missing from the list? What else should be measured?

## **Part 3- Implementing self-reported measures questionnaires in maternity care routine (5-10 mins)**

Image that for health professionals to follow your health status and provide you better care, you will be asked to regularly respond to this kind of self-reported questionnaire during the pregnancy and postpartum until baby is six months or one year old.

- What do you concern and expect?
- Are you willing to regularly respond to this kind of self-reported questionnaire to report your health status, feelings, and experiences, etc.? What could motive you to answer the questions? what could be the obstacles?
- How should the measures and data be used?

## **Ending (3 mins)**

Thank you very much for your time! You provided us very useful information.

Wish you a happy time! Enjoy! …
